# Cross-kingdom regulation of gene expression in giant pandas via plant-derived miRNA

**DOI:** 10.3389/fvets.2025.1509698

**Published:** 2025-02-28

**Authors:** Helin Tan, Chengdong Wang, Feng Li, Yue Peng, Jiacheng Sima, Ying Li, Linhua Deng, Kai Wu, Zhongxian Xu, Zejun Zhang

**Affiliations:** ^1^Key Laboratory of Southwest China Wildlife Resources Conservation (Ministry of Education), College of Giant Panda, China West Normal University, Nanchong, China; ^2^Liziping Giant Panda's Ecology and Conservation Observation and Research Station of Sichuan Province, China West Normal University, Nanchong, China; ^3^China Conservation and Research Center for the Giant Panda, Key Laboratory of SFGA on the Giant Panda, Chengdu, China; ^4^Guangdong Provincial Key Laboratory of Animal Molecular Design and Precise Breeding, School of Animal Sciences and Technology, Foshan University, Foshan, China

**Keywords:** exosome, plant-derived miRNA, cross-kingdom regulatory, giant panda, dietary transition, bamboo

## Abstract

Giant pandas (*Ailuropoda melanoleuca*) belong to the order Carnivora, but they mainly feed on bamboo, and their unique dietary adaptability has always been the focus of research. Recent research indicates that plant-derived microRNAs (miRNAs) can be delivered to animal organisms via exosomes and exert cross-kingdom regulatory effects on gene expression. To explore the role of plant-derived miRNAs in the dietary adaptation of giant pandas, we collected peripheral blood samples from three groups of pandas: juvenile females, adult females, and adult males—and extracted exosomes from the blood for small RNA sequencing. Additionally, three types of bamboo (shoots, stems, and leaves) consumed by the pandas were sampled for miRNA sequencing. Through comparative analysis, we identified 57 bamboo-derived miRNAs in the extracellular exosomes of giant panda peripheral blood. Gene Ontology (GO) and Kyoto Encyclopedia of Genes and Genomes (KEGG) functional enrichment analyses of the target genes for these miRNAs revealed their involvement in various pathways, including taste and olfactory signal transduction, digestion and absorption, and hormonal signal transduction. Furthermore, we found that plant-derived miRNAs can modulate dopamine metabolism in giant pandas, thereby influencing their food preferences. This study shows that plant-derived miRNAs can enter the bloodstream of giant pandas and exert cross-kingdom regulatory effects, potentially playing a vital role in their dietary adaptation process.

## 1 Introduction

Exosomes are nano-sized vesicles (40–100 nm) released by various cell types into the extracellular space and act as physiological carriers of microRNAs (miRNAs) (1). miRNAs are small noncoding RNAs approximately 18–25 nucleotides in length, and they exert post-transcriptional regulatory effects by pairing complementarily with specific sequences in the 3′ untranslated regions of target mRNAs (2). miRNAs regulate essential biological processes, including apoptosis, metabolism, immune responses, hormone signaling, cell proliferation, and differentiation (3). While it is widely accepted that miRNAs predominantly function to negatively regulate gene expression, some studies indicated that they can oscillate between suppression and activation, mediating gene activation during the G1/G0 cell cycle arrest (4). Moreover, miRNAs can induce gene expression by activating target genes (5). Previous research has shown that plant-derived miRNAs can enter animal bodies and perform gene regulatory functions (6). The stability of mature plant miRNAs is due to their inherent resistance to degradation, which is maintained during mammalian circulation and after boiling. This stability is further enhanced by encapsulation in micro vesicles or exosomes during transport, protecting them from degradation (7). Plant-derived miRNAs can be absorbed through the diet and are detectable in animal serum and plasma. For instance, miRNAs from ginger can enter the animal gut through consumption and target intestinal microbiota, influencing the progression of colitis (8). Evidence demonstrates that plant-derived miRNAs play crucial roles in antiviral responses, antitumor activities, apoptosis inhibition, immune modulation, and the regulation of intestinal function in mammals (7).

The giant panda (*Ailuropoda melanoleuca*) serves as a flagship species for global biodiversity conservation. Its dietary habits are highly specialized, featuring a gastrointestinal tract typical of carnivores while exhibiting traits characteristic of extreme herbivores (9). The diet predominantly consists of fibrous bamboo (9) which has an exceptionally low utilization rate (10). Nevertheless, the giant panda has evolved a distinct set of features to adapt to its bamboo-based diet. Morphologically, the giant panda possesses a pseudo-thumb at the base of its forelimb, enabling it to grasp bamboo with its other five digits (11). Its skull consists of dense, compact bones and, compared to other bears, feature extremely expanded zygomatic arches (12) and a well-developed mandible structure (13). These structures, along with its flat teeth, are well suited for crushing and chewing tough bamboo (14).

Ecologically, the giant panda has achieved long term coexistence with other wildlife in its habitat through its unique bamboo-feeding habits and selective consumption of different bamboo parts (15). In the wild, giant pandas exhibit seasonal rhythms in their choice of bamboo and habitat, which help mitigate seasonal energy constraints (16–18). Behaviorally, giant pandas reduce their activity levels while increasing both their feeding intake (19) and feeding time (10) to maximize nutrient extraction from low nutrition bamboo. Physiologically, the gut microbiota of giant pandas predominantly consists of Firmicutes, which enhances the digestion and nutrient utilization of bamboo (20). This unique gut microbiota is also enriched in genes related to starch and sucrose metabolism, fulfilling the pandas’ daily energy requirements (21, 22). Additionally, the intestinal lining of giant pandas has evolved numerous mucus secreting glands to aid in the digestion of high fiber food and to prevent gastrointestinal damage (23). Genetically, the giant panda’s umami taste receptor gene TAS1R1 is pseudogenized, adapting to the low umami content of bamboo (24, 25). Furthermore, a mutation in the DUOX2 gene results in decreased thyroid hormone levels, which leads to reduced energy expenditure (26).

Wild giant pandas primarily feed on bamboo. However, we provide captive giant pandas with a variety of supplementary foods, including apples, carrots, and bread-like products made from wheat and corn. This diet is designed to ensure nutritional balance and promote overall health. Research has shown that a common plant miRNA, MIR156, found in soybeans, wheat, and corn, regulates intestinal cell proliferation, health, and development. It helps maintain intestinal epithelial homeostasis and prevents colitis (27, 28). The cross-kingdom regulation of gene expression by plant miRNA may also explain why giant pandas, which possess a carnivorous gastrointestinal tract, can adapt to the herbivorous diet. Investigations into the causes of this dietary transition have primarily focused on genetic mutations in giant pandas (25) and historical environmental changes. While researchers have made progress in understanding how giant pandas adapt to a bamboo diet (29), the role of dietary plant-derived miRNA in this dietary shift and adaptation remains unexplored.

The transition of giant pandas from a carnivorous diet to a bamboo-based diet, along with their subsequent adaptation to this dietary shift, has long been a focal point of scientific inquiry. This study aims to investigate whether plant-derived miRNA can enter the giant panda’s body and regulate gene expression, thereby facilitating a more effective adaptation to a bamboo-based diet.

## 2 Materials and methods

### 2.1 Focal animals

All giant pandas involved in this study were healthy and maintained by the China Conservation and Research Center for the Giant Panda (Supplementary Table S1). We collected blood samples during routine health check-ups of the giant pandas, ensuring that no additional blood collection procedures were performed solely for this study. This approach minimized any potential extra stress or disturbance to the giant pandas.

### 2.2 Peripheral blood collection and exosome miRNA sequencing in giant pandas

Giant panda blood samples were obtained from May to June 2022, with each giant panda providing a 10 mL blood sample. Due to the limited blood volume available from the juvenile female, we collected samples three times during the sampling period and combined them into one sample for subsequent analysis (Supplementary Table S1). Following to the manufacturer’s instructions, we isolated and enriched extracellular vesicles from the collected peripheral blood of the giant pandas. We extracted total RNA using the MagCapture™ Exosome Isolation Kit PS (Wako Pure Chemical Industries, Japan). We then analyzed the exosome samples for total RNA quantity and fragment distribution using the highly sensitive Agilent 2100 Bioanalyzer. After verifying the sample quality, we employed the Small RNA Sample Prep Kit to construct the library. We used the total RNA as the starting material, taking advantage of small RNA’s unique 3′ and 5′ structural characteristics (with a complete phosphate group at the 5′ end and a hydroxyl group at the 3′ end). We ligated adapters directly to both ends of the small RNA, followed by reverse transcription to synthesize cDNA. After PCR amplification, we separated the target DNA fragments using PAGE gel electrophoresis and recovered the resulting fragments from the gel to obtain the cDNA library.

We prepared seven small RNA libraries derived from the peripheral blood exosomes of giant pandas. After constructing the libraries, we conducted preliminary quantification using Qubit 2.0. We diluted the library to 1 ng/μL. Subsequently, we assessed the insert size of the library using the Agilent 2100. Once the insert size met expectations, we performed accurate quantification of the library’s effective concentration using Q-PCR (with an effective concentration >2 nM) to ensure library quality. Upon passing quality control, we pooled the libraries based on effective concentration and target sequencing data requirements before subjecting them to Illumina SE50 sequencing, with a single-end sequencing length of 50 bp.

### 2.3 miRNA sequencing of bamboo derived from panda diet

During the blood sampling, the giant pandas consumed bamboo from three species and various plant parts. As a result, we collected samples from the three bamboo species and their different parts consumed during the blood sampling period for sequencing: bamboo stem (BJZ1), bamboo shoot (JZS1) and bamboo leaf (FZY1). We immediately preserved the bamboo samples at −80°C and stored them on dry ice before transporting them to Novogene Bioinformatics Technology Co., Ltd.^1^ for miRNA extraction and sequencing. After acquiring clean reads, we mapped the small RNA sequencing data to the reference genome using Bowtie (30) alignment software. We compared the aligned reads with plant miRNA sequences from the miRBase database to determine the sequence matching across different regions in each sample. We identified known miRNAs and characterized and annotated their expression levels, sequences, lengths, and secondary structures. This analysis provided insights into the types of known miRNAs present in the bamboo samples.

### 2.4 Data analysis

To obtain high-quality reads, we performed initial filtering of the raw data in FASTQ format. We removed low-quality reads, defined as those where the number of bases with a quality score ≤ 20 constitutes more than 30% of the entire read. We also excluded reads with more than 10% of bases designated as ‘N’ (indicating undetermined base information), reads contaminated with 5′ adapters, reads lacking 3′ adapter sequences and insertion fragments, and reads containing polyA/T/G/C sequences. This process generated clean reads. The adapter information for the small RNA included the RNA 5′ Adapter (RA5): 5′-GTTCAGAGTTCTACAGTCCGACGATC-3′, and the RNA 3′Adapter (RA3): 5′-AGATCGGAAGAGCACACGTCT-3′. We then analyzed the length distribution of clean reads for each sample. We mapped the length-filtered small RNA to reference sequences using Bowtie (30) and compared the results with plant database sequences in miRBase. Finally, we compared the obtained miRNA with that of bamboo to identify the plant-derived miRNA present in the giant panda.

### 2.5 Prediction of target genes and functional enrichment analyses

For animal-derived miRNA, we initially performed target gene prediction using miRanda (31). This tool identifies the top 10 candidate target genes within the 3′UTR of each miRNA. When multiple miRNAs correspond to the same target site, miRanda employs a greedy algorithm to select the pair with the highest score and lowest free energy. Subsequently, we used the RNAhybrid (32) prediction algorithm to exclude the formation of intramolecular, miRNA-miRNA, and miRNA-target gene dimers. We then detected the optimal target sites based on the binding energy between miRNAs and target genes. The intersection of results from both software tools constituted the final prediction for miRNA target genes. For plant-derived miRNA, we conducted target gene prediction using the TargetFinder (33) software to analyze. Known miRNAs and establish their target gene relationships.

To facilitate the selection of differentially expressed miRNAs, we quantified and normalized the expression levels of known miRNAs in each sample using TPM (34). The input data for differential miRNA expression analysis consisted of read count data from miRNA expression level assessments. We conducted the analysis of samples with biological replicates using DESeq (30), based on a negative binomial distribution. We set the default criteria for identifying differentially expressed miRNAs at padj <0.05, which resulted in the detection of differentially expressed miRNAs. We subjected the target genes of differentially expressed miRNAs, as well as those of all known miRNAs, to GO and KEGG pathway enrichment analyses using the DAVID website. This process aims to identify biologically significant functions and pathways for further investigation.

## 3 Results

### 3.1 Plant-derived miRNAs in blood exosomes of giant pandas

To detect whether the blood exosomes of giant panda contain plant-derived miRNA, we collected peripheral blood samples from three adult male giant pandas (ME), three adult female giant pandas (FE), and one juvenile female giant panda (FY) for exosome enrichment, miRNA extraction and sequencing (Supplementary Table S1). We obtained a total of 82,148,047 high-quality SE50 reads across seven small RNA libraries, with an average GC content of 52.25% across all samples. The reads achieved a Q20 score of at least 98.82% (Supplementary Table S2), confirming that the sequencing data quality is sufficient to ensure accurate subsequent analyses. We compared the sequenced miRNAs to the universal plant miRNA database in miRBase, which led to the annotation of 57 mature plant miRNAs, along with their corresponding annotation names and mature sequences (Supplementary Table S3).

To determine whether the miRNAs detected in the blood of giant pandas were derived from their food, bamboo stem (*Phyllostachys bissetii*, BJZ1), bamboo shoot (*Phyllostachys sulphurea*, JZS1) and bamboo leaf (*Chimonobambusa quadrangularis*, FZY1), the main food of giant pandas, were collected during the blood collection period for miRNA extraction and sequencing. For the three bamboo samples, Illumina high-throughput sequencing produced 33,482,573 high-quality reads. We quantified and analyzed the total clean reads and the corresponding matched reads (Supplementary Table S4). From these bamboo samples, we annotated 10,514 mature plant miRNAs (Supplementary Table S5). Notably, all 57 plant miRNAs identified in giant panda blood matched those found in the bamboo samples, suggesting their likely origin from bamboo.

**Figure 1:**
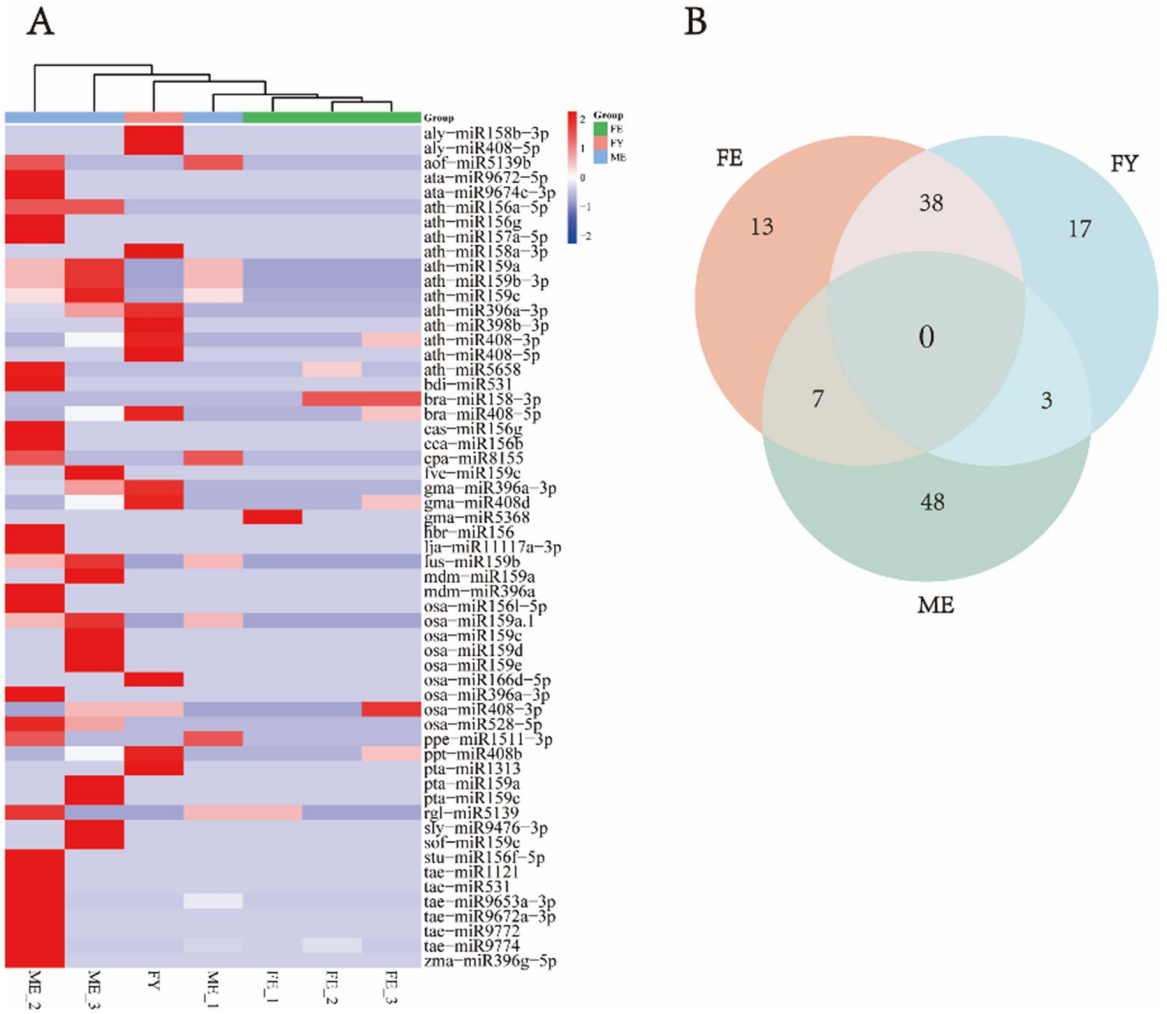
Types and abundances of plant-derived miRNA identified in exosomes from peripheral blood of giant pandas. **(A)** Heatmap of miRNA clustering in exosomes from giant panda blood. **(B)** Venn diagram illustrating the differential expression of miRNAs in exosomes derived from giant panda blood.

Since there are no specific miRNA databases for the bamboo species mentioned above, these plant-derived miRNAs from giant panda blood were annotated in the universal plant database, including apple miRNAs such as mdm-miR159a and mdm-miR396a; wheat miRNAs, including tae-miR1121, tae-miR531, tae-miR9653a-3p, tae-miR9672a-3p, tae-miR9772, and tae-miR9774; and maize miRNA: zma-miR396g-5p (Figure 1A). Notably, tae-miR9774, tae-miR531, and tae-miR9653a-3p showed particularly high abundance. The diet of captive giant pandas now includes not only bamboo but also apples, carrots, and bread-like products containing wheat and maize, such as steamed buns, suggesting that these miRNAs are derived from various ingested foods beyond bamboo. These findings suggest that food-derived plant miRNAs are present in the peripheral blood of giant pandas.

We also compared the differences in the composition of plant-derived miRNAs between different groups of giant pandas, and observed that plant-derived miRNAs are more prevalent and diverse in the FY and ME groups (Figure 1A). No miRNAs were detected in all three groups simultaneously (Figure 1B). A higher overlap was observed between the FE and FY groups, indicating greater co-expression of plant-derived miRNAs within the same sex. The ME group displayed the largest number of uniquely existing miRNAs, suggesting a higher specificity of miRNAs in adult male giant pandas (Figure 1B).

### 3.2 Functional enrichment results of differential miRNA target genes

#### 3.2.1 Adult versus juvenile female giant pandas

**Figure 2:**
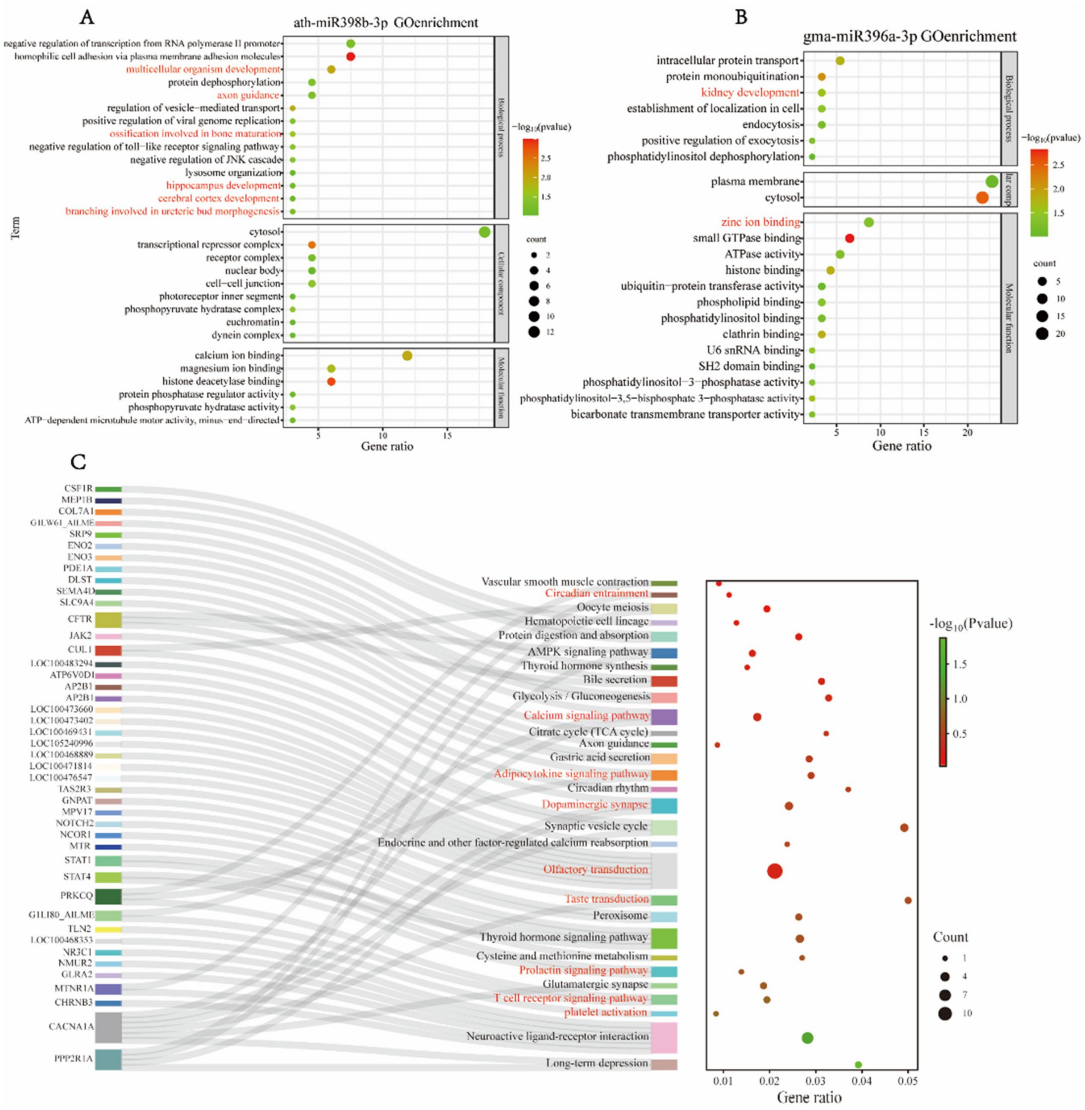
GO enrichment and KEGG pathway analysis of differentially expressed miRNA target genes in FE and FY. **(A,B)** Enrichment analysis of target genes for **(A)** ath-miR398b-3p and **(B)** gma-miR396a-3p. **(C)** KEGG pathway enrichment analysis of differentially expressed miRNA target genes in FE and FY.

Due to differences in dietary composition among giant pandas of varying ages, we established a group for comparative analysis: FE vs. FY, to investigate the differences in miRNA expression within peripheral blood exosomes between adult and juvenile giant pandas, and to elucidate the functions of these differential miRNAs. Gene Ontology (GO) functional enrichment analysis of the target genes of differential miRNAs in the FE vs. FY group revealed that ath-miR398b-3p was exclusively detected in the blood of FY (Figure 2A). The GO enrichment analysis of its target genes indicated involvement in key biological processes related to juvenile development, including multicellular organism development, ossification involved in bone maturation, branching involved in ureteric bud morphogenesis, hippocampus development, cerebral cortex development, and axon guidance. Additionally, significant biological functions such as calcium ion binding and magnesium ion binding were identified in the enrichment results (Figure 2A). We also detected gma-miR396a abundantly in the blood of FY, and the target genes of this miRNA were associated with critical biological processes, such as kidney development, and key biological functions, including zinc ion binding (Figure 2B).

Kyoto Encyclopedia of Genes and Genomes (KEGG) functional enrichment analysis of miRNAs revealed that ata-miR9672-5p, rgl-miR5139, and ath-miR5658 were exclusively detected in the blood of adult giant pandas, and the target genes of these miRNAs were found to regulate circadian entrainment in the species (Figure 2C). Several miRNAs, including ath-miR159b-3p, ath-miR159c, ath-miR159a, and osa-miR159a.1, were more prevalent in male and juvenile individuals. Their target genes were involved in platelet activation (Figure 2C). The target genes of ppt-miR408b, osa-miR408-3p, gma-miR408d, and ath-miR398b-3p regulate the T cell receptor signaling pathway, and while these miRNAs are present in all giant pandas, they are more abundant in juveniles. Bra-miR158-3p was exclusively detected in female individuals, with its target genes significantly enriched in the prolactin signaling pathway, as indicated by KEGG analysis (Figure 2C). The target genes of tae-miR9653a-3p and hbr-miR156 were associated with the taste transduction pathway.

Furthermore, multiple miRNAs had target genes enriched in the olfactory transduction pathway, and several miRNAs targeted genes involved in regulating the dopaminergic synapse pathway (Figure 2C). The target genes of ppt-miR408b, osa-miR408-3p, gma-miR408d, osa-miR396a-3p, and gma-miR396a-3p regulate the adipocytokine signaling pathway (Figure 2C). Increased adipocyte volume and number are positively correlated with leptin production, and leptin’s effects are mediated through JAK kinase, STAT3 phosphorylation, and nuclear transcriptional effects. JAK-mediated phosphorylation of SHP-2 regulates giant panda cell growth and proliferation, which is part of the MAPK signaling pathway. The target genes of tae-miR9653a-3p, ath-miR398b-3p, and gma-miR396a-3p modulate the calcium signaling pathway. These miRNAs are more prevalent in juvenile giant pandas, potentially contributing to skeletal morphology development and environmental information processing. Additionally, the target genes of ath-miR398b-3p and gma-miR396a-3p are involved in the AMPK signaling pathway (Figure 2C). The correspondence between miRNAs and their target genes is detailed in Supplementary Figure S1.

#### 3.2.2 Adult female and male giant pandas

**Figure 3:**
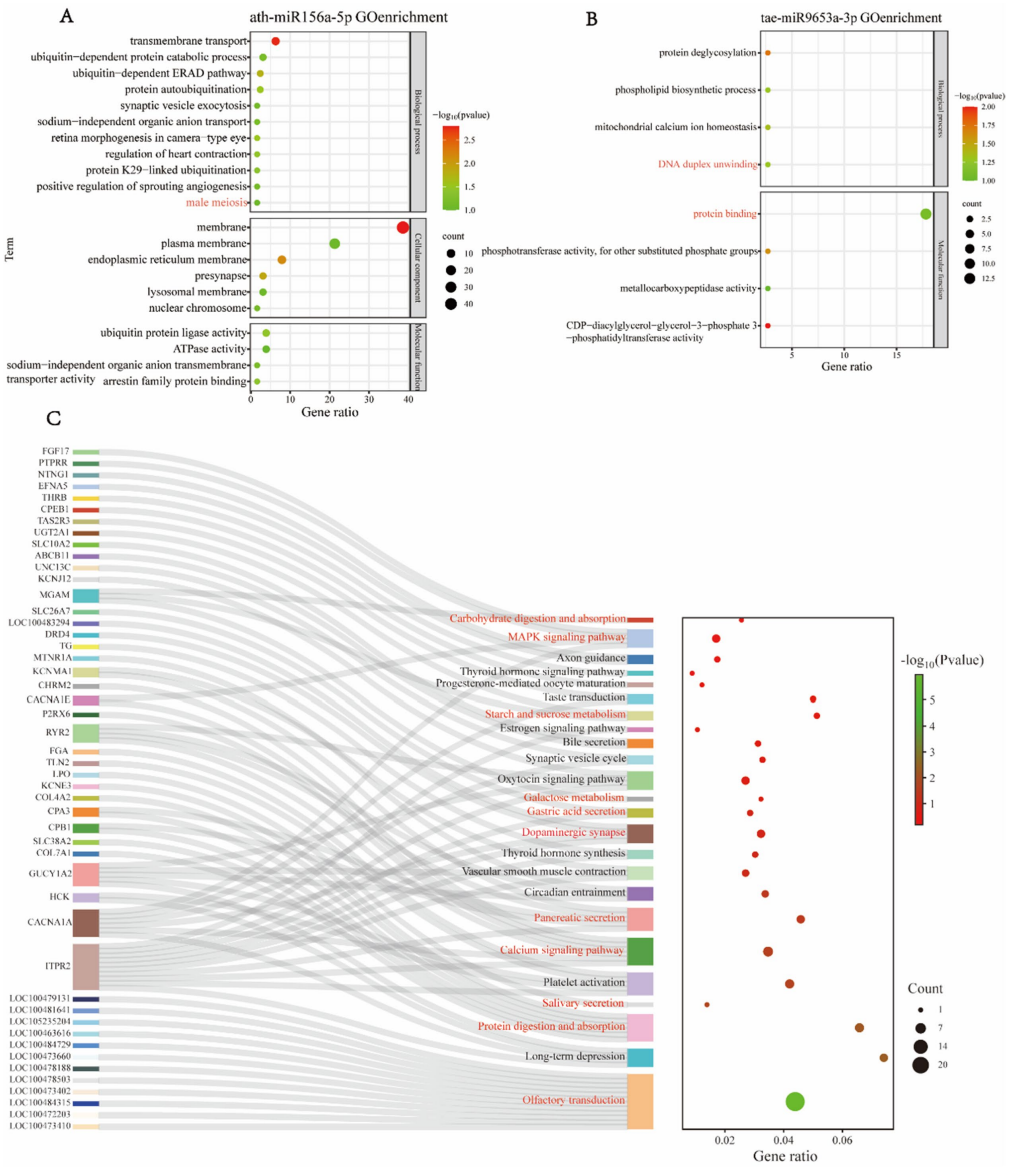
GO enrichment and KEGG pathway analysis of differentially expressed miRNA target genes in ME and FE. **(A,B)** Enrichment analysis of target genes for ath-miR156a-5p **(A)** and tae-miR9653a-3p **(B)**. **(C)** KEGG pathway enrichment analysis of differentially expressed miRNA target genes in ME and FE.

Due to potential genetic expression discrepancies between sexes, we established a group for comparative analysis: FE vs. ME, to investigate the differences in miRNA expression within peripheral blood exosomes between female and male giant pandas, and to elucidate the functions of these differential miRNAs. GO functional enrichment analysis of the target genes of differential miRNAs in the ME vs. FE group revealed that ath-miR156a-5p was detected exclusively in the blood of male giant pandas and was absent in females. GO enrichment analysis of its target genes highlighted their association with male meiosis (Figure 3A). Additionally, we performed GO enrichment analysis on the target genes of tae-miR9653a-3p, which revealed significant associations with the biological process of DNA duplex unwinding and the biological function of protein binding (Figure 3B).

KEGG functional enrichment analysis of miRNAs revealed that the target genes regulated by tae-miR9653a-3p and hbr-miR156 influenced the taste transduction pathway, similar to what was observed in the FE vs. FY group. Additionally, the target genes of various miRNAs were implicated in the olfactory transduction pathway in giant pandas. These miRNAs, detected in the blood of male, female, and juvenile giant pandas, had target genes associated with the regulation of dopaminergic synapses (Figure 3C). Moreover, multiple miRNAs had target genes involved in the MAPK signaling pathway (Figure 3C), which regulates key cellular processes such as growth, differentiation, environmental stress responses, and inflammation. Various miRNAs also targeted genes related to digestive, absorptive, and metabolic processes, including starch and sucrose metabolism, carbohydrate digestion and absorption, galactose metabolism, gastric acid secretion, salivary secretion, protein digestion and absorption, and pancreatic secretion. Several miRNAs had target genes associated with the calcium signaling pathway (Figure 3C). The correspondence between miRNAs and their target genes is detailed in Supplementary Figure S1.

### 3.3 Functional enrichment of individual plant-derived miRNA target genes

**Figure 4:**
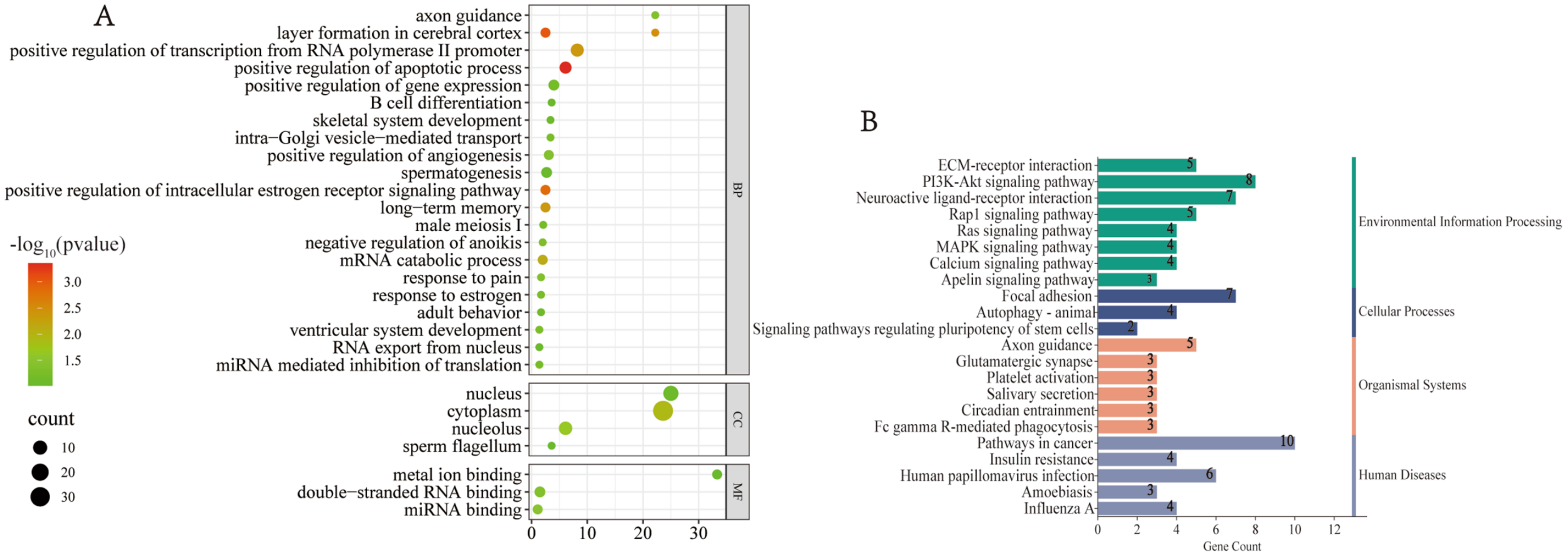
Functional enrichment of the target genes of individual miRNAs. **(A)** Bubble plot of significant GO terms. **(B)** Bar chart of significant KEGG pathways.

Ultimately, in addition to performing functional enrichment analysis on the target genes of the differentially expressed miRNAs, GO functional enrichment was also conducted for individual miRNAs. Significant GO terms were selected and visualized for inclusion in Figure 4A. This analysis revealed several miRNAs with specific expression patterns, such as: osa-miR528-5p was detected exclusively in the blood of adult male giant pandas. The biological processes enriched in the target gene GO terms of this miRNA include male meiosis I and the positive regulation of angiogenesis. ath-miR159c was detected solely in the blood of male giant pandas, with its target genes enriched in GO terms related to sperm flagellum formation and B cell differentiation. aly-miR158b-3p was detected only in the blood of female giant pandas, and its target gene GO enrichment revealed important biological processes such as response to estrogen and the positive regulation of intracellular estrogen receptor signaling pathways. These findings suggest that miRNAs may specifically regulate the reproductive processes of giant pandas, with only those miRNAs that modulate gene expression unique to the species being retained in the body. Moreover, other miRNAs exhibited target gene GO enrichment in several significant biological functions, including the negative regulation of anoikis, B cell differentiation, adult locomotor behavior, metal ion binding, long-term memory, and axon guidance. Notably, the negative regulation of anoikis refers to any process that inhibits, prevents, or reduces the frequency, rate, or extent of anorexia, which may potentially prevent anorexic behavior in giant pandas and ensure sufficient bamboo intake on a daily basis. These results underscore the involvement of miRNA targeted genes in various life activities of giant pandas, including the regulation of reproduction and behavior.

Subsequently, the target genes of individual miRNAs were subjected to KEGG functional enrichment analysis, and key pathways were selected for visualization, as shown in Figure 4B. The pathways associated with the target genes of these miRNAs predominantly involve environmental information processing, cellular processes, organic systems, and diseases. Notable enriched pathways include axon guidance, neuroactive ligand–receptor interaction, platelet activation, salivary secretion, circadian entrainment, calcium signaling, FcγR mediated phagocytosis, MAPK signaling, and Ras signaling. Among these, the MAPK signaling pathway regulates several critical cellular physiological and pathological processes, such as cell growth, differentiation, stress adaptation to environmental stimuli, and inflammatory responses. The Ras signaling pathway is one of the pivotal intracellular signal transduction mechanisms, participating in numerous biological processes, including cell growth, differentiation, apoptosis, and metabolism. Phagocytosis is primarily triggered by the aggregation of FcγR at the contact sites between leukocytes and phagocytic particles. It is a vital component of the innate immune response, playing a key role in the ingestion and destruction of infectious pathogens as part of the host defense mechanism. These findings suggest that miRNAs are involved in various essential biological activities, including immunity and behavior, in the giant panda.

## 4 Discussion

### 4.1 Variations in plant-derived miRNA in peripheral blood exosome of giant pandas across ages and genders

In this study, we analyzed exosomes isolated from the peripheral blood of three groups of giant pandas using small RNA sequencing. Our research aimed to investigate the cross-kingdom regulatory effects of exogenous plant-derived miRNA on gene expression in giant pandas. Prior to this study, researchers had reported only the expression profiles of miRNA in giant panda blood and milk (35–37), as well as in five different tissues (38). These previous studies primarily focused on detecting endogenous miRNAs in giant pandas and did not elucidate the cross-kingdom effects of plant-derived miRNA present in giant panda exosomes on the gene expression of the giant pandas themselves.

Upon analyzing miRNAs from the peripheral blood exosomes of giant pandas, we observed variability in miRNA profiles among different individuals. We identified a greater number of uniquely present miRNAs in the blood of adult male giant pandas. The heatmap of miRNA in the peripheral blood exosomes of giant pandas shows that both the types and quantities of miRNA are greater in adult male and juvenile female giant pandas compared to adult female giant pandas. It was found by Yang et al. that the expression levels of endogenous miRNAs in giant pandas are significantly higher in juvenile individuals compared to adult pandas (36), these endogenous miRNAs are involved in the host immune response and the Ras signaling pathway, our research has revealed that certain plant-derived miRNAs were detected exclusively in the blood of juvenile giant pandas; these miRNAs target genes involved in growth, development, morphological formation, and the regulation of platelet activation. This finding suggests that miRNAs may play a role in the growth and developmental processes of juvenile giant pandas, plant-derived miRNA is likely retained due to its role in growth and development, with these miRNAs acquired from food and maternal milk (37). Additionally, certain miRNAs were also detected only in the blood of male giant pandas, with their target genes implicated in spermatogenesis and reproductive regulation. It is suggested that miRNAs may play a role in regulating the mechanisms of sperm cryotolerance (39), consistent with our findings. Conversely, some miRNAs were found exclusively in the blood of female giant pandas, with their target genes associated with female reproductive signaling pathways, oogenesis, and the regulation of reproductive behaviors. This indicates that miRNA mediated regulatory functions in giant pandas vary with age and sex; only those miRNAs capable of modulating specific gene expressions are retained within the organism. It is evident that the impact of miRNAs from bamboo on the gene expression of giant pandas is both long term and persistent. During infancy, miRNAs are acquired from maternal milk, where they regulate growth and development. As the panda matures and bamboo becomes incorporated into its diet, miRNAs derived from bamboo continue to modulate gene expression, facilitating the panda’s adaptation to a bamboo-based diet.

### 4.2 Plant-derived miRNA can enter giant pandas and exercise post-transcriptional regulation

We conducted small RNA sequencing and annotation of the bamboo consumed by giant pandas. By comparing these sequences with mature plant miRNAs identified and annotated in the exosomes of giant panda peripheral blood, we determined that 57 miRNAs present in the giant panda’s blood are derived from bamboo and other dietary sources. This finding indicates that these miRNAs originate solely from dietary intake and suggests that plant-derived miRNAs are remarkably stable. They can withstand enzymatic digestion in the gastrointestinal tract, traverse the intestinal barrier, and enter systemic circulation and various organs (40). Subsequently, these miRNAs regulate endogenous targets in a cross-kingdom manner, thereby influencing the cellular systems of their receptors (41). Once pre-miRNAs are processed into mature miRNAs, they can target one or multiple genes. Conversely, a single mRNA target can be regulated by multiple miRNAs (42). Our findings also indicate that phenomenon (Supplementary Figure S1).

The miRNA mediated regulation of target genes is a complex process. Generally, miRNAs exert negative regulation on gene expression; however, studies have indicated that miRNAs can not only suppress gene expression but also induce it (43). In our study, ath-miR398b-3p has been identified as targeting crucial gene pathways involved in cerebral cortex development, ossification related to bone maturation, hippocampus development, and inorganic ion binding in giant pandas (Figure 2A). This miRNA may regulate these biological processes, thereby influencing body morphology, nervous system development, and the absorption and utilization of inorganic ions in juvenile giant pandas. Furthermore, the target genes of ppt-miR408b are enriched in biological functions associated with the positive regulation of angiogenesis (Supplementary Figure S2G). Multiple miRNA target genes were functionally enriched in the platelet activation pathway, as illustrated in Figure 2C. Notably, aly-miR158b-3p was detected exclusively in the blood of female giant pandas. The GO enrichment analysis of its target genes revealed biological processes critical for response to estrogen and positive regulation of intracellular estrogen receptor signaling pathways (Supplementary Figure S2D). If the miRNA functions solely in a negative regulatory capacity to induce gene silencing, then the consumption of bamboo would be detrimental to giant pandas and would fail to support their bamboo specialization.

### 4.3 Plant-derived miRNA may target and regulate the appetite reward system in giant pandas

Research indicates that the propensity to engage in or sustain eating behavior is influenced by the taste of food, the gut’s response to food components, and the potential impact of reward pathways in the brain (44, 45). Animal studies have demonstrated that both opioids and dopamine are involved in the appetite reward circuitry associated with food intake behavior (46, 47). Recent studies have highlighted the critical role of dopamine in stimulus reward learning behavior (48). Dopamine metabolism in giant pandas is involved in food selection processes, with both excessive and deficient levels of dopamine exerting profound effects on feeding behavior. The dopamine metabolism system in giant pandas may be relatively weak (49). Certain components in bamboo might influence dopamine metabolism in giant pandas; these components may stimulate the appetite-reward circuitry in giant pandas and play a role in their bamboo consumption context (49).

The target genes of tae-miR9653a-3p, stu-miR156f-5p, gma-miR5368, ath-miR158a-3p, aly-miR158b-3p, ath-miR156g, ath-miR156a-5p, and cas-miR156g are predicted to target the dopamine synaptic pathway in giant pandas (Figures 2C, 3C). Dopamine, a crucial and typical slow neurotransmitter in the mammalian brain, modulates various functions, including motor activity, motivation and reward, learning and memory, and endocrine regulation. The target gene of ath-miR156g is DRD4, a type of dopamine receptor. It is suggested that this miRNA may interact with its target gene. These miRNAs may bind to target mRNAs, leading to gene silencing or mRNA degradation. This process could weaken the dopamine metabolism system in giant pandas, thereby modulating the metabolic pathways of dopamine. Consequently, the slower metabolism of dopamine during bamboo consumption may enhance pleasurable sensations, influencing giant pandas’ dietary preference for bamboo.

### 4.4 Adoption of a bamboo-based diet in giant pandas may be due to post-transcriptional regulation of physiological processes by plant-derived miRNA

Fifty-seven identified plant-derived miRNAs regulate various physiological processes in giant pandas, including growth and development, biological rhythms, behavior, immune responses, reproductive systems, energy metabolism, circulatory systems, hormonal signaling, taste and olfactory signaling, locomotion, and digestion and absorption. Plant-derived miRNAs can target the taste and olfactory pathways in giant pandas. Specifically, tae-miR9653a-3p and hbr-miR156 target genes involved in the taste transduction pathway (Figure 2C). The target gene of hbr-miR156, ENSAMEG00000019797, encodes Taste Receptor Member 3 (TAS2R3). TAS2R3 mediate bitterness perception (50). For herbivores, retaining sensitivity to bitterness constitutes a vital survival strategy. In contrast to other carnivores, giant pandas possess a more comprehensive TAS2R library, which allows them to detect bitter substances in bamboo and adapt to a diet mainly composed of bamboo (51). The miRNAs in bamboo regulate the expression of TAS2R3, rendering giant pandas more acutely sensitive to bitterness. This enables them to identify toxins in foods such as bamboo and make safer choices regarding their diet.

Various plant-derived miRNAs target genes associated with olfactory transduction in giant pandas. It is hypothesized that as giant pandas age and bamboo consumption increases, these miRNAs accumulate, thereby modulating gene expression pathways and aiding in their adaptation to the taste of bamboo. Additionally, plant-derived miRNAs may influence the olfactory system, enabling giant pandas to discern the nutritional value and edibility of bamboo, which facilitates the selection of the freshest and most nutritious bamboo for consumption. Osa-miR166d-5p targets and regulates the negative regulation of anoikis (Supplementary Figure S2C). This miRNA may modulate gene expression in giant pandas, preventing anorexic responses and enabling the adaptation of their digestive systems—originally suited for carnivorous diets—to a bamboo-based diet with low nutritional value. This adaptation ensures that giant pandas consume sufficient bamboo daily to meet their energy requirements (52), this can also be considered as a strategy through which miRNAs modulate the behavior of giant pandas, facilitating their adaptation to a bamboo-based diet. Interestingly, tae-miR9653a-3p has been found to simultaneously regulate dopamine, olfactory, and taste transduction pathways in giant pandas. Notably, this miRNA is derived from wheat, which may suggest that giant pandas, in addition to bamboo, might also consume wheat-containing foods like ‘wowo tou’ (bread-like products containing wheat and corn), potentially mediated by miRNAs present in the food. This observation further supports the hypothesis that the selection of bamboo as the primary diet by giant pandas is associated with the regulation of these three pathways. Thus, the cross-kingdom gene expression by plant-derived miRNAs may underlie the dietary transition observed in giant pandas.

Various miRNAs target genes involved in the digestion, absorption, and metabolism processes of the giant panda, thereby sustaining their vital functions. Additionally, miRNAs derived from bamboo regulate angiogenesis and immunity in the giant panda, as well as the absorption and utilization of inorganic ions. These regulatory functions of miRNAs enable the giant panda to adapt to the complex survival conditions in the wild while consuming nutrient-poor bamboo. Several miRNAs also modulate adipocytokine signaling pathways, MAPK signaling pathways, and AMPK signaling pathways. The MAPK pathway influences cell growth and proliferation, while the activation of the AMPK pathway inhibits energy consuming biosynthetic processes, such as protein, fatty acid, and glycogen synthesis. Concurrently, it stimulates ATP producing catabolic pathways, including fatty acid oxidation and glycolysis. This mechanism may explain why the giant panda, despite consuming nutritionally poor bamboo, can attain a relatively robust physique. Historically, wild giant pandas have faced reproductive challenges, such as difficulties in estrus, conception, cub rearing, and survival. However, captive populations have effectively addressed these challenges (52). Some miRNAs are exclusively present in either males or females and specifically target and regulate processes related to the formation of reproductive cells, responses to sex hormones, and sex hormone signaling pathways in the giant panda. This indicates that miRNAs derived from bamboo also play a regulatory role in the reproductive processes of the giant panda. However, the regulatory effects of miRNAs can be either positive or negative. It remains to be determined whether the miRNAs present in bamboo contribute to the reduced reproductive capacity of the giant panda or if the inherently low reproductive capacity is mitigated by the regulation of related miRNAs from bamboo, thereby allowing for the maintenance of population viability.

## 5 Conclusion

The dietary shift and adaptation of the giant panda have long intrigued researchers. Previous studies have predominantly focused on the macro and micro-level changes within the giant panda itself. This dietary shift represents a bidirectional interaction with their food, where miRNAs from bamboo may facilitate the adaptation to a bamboo-exclusive diet. Based on the theory of plant-derived miRNA mediated cross-kingdom regulation of animal gene expression, we found that plant-derived miRNAs can regulate dopamine metabolism pathways, as well as taste and olfactory signal transduction pathways. It is plausible that these plant-derived miRNAs mediate the transition of the giant panda from a carnivorous to a bamboo-based diet. This study presents a novel approach to investigating the dietary changes and adaptive mechanisms of the giant panda.

## Data Availability

The datasets presented in this study can be found in online repositories. The names of the repository/repositories and accession number(s) can be found at: https://www.ncbi.nlm.nih.gov/, PRJNA1134401.

## Footnotes

^1^ https://www.novogene.cn
