# Supplementary material for: Cross-kingdom regulation of gene expression in giant pandas via plant-derived miRNA
Source: Front Vet Sci. 2025 Feb 28;12:1509698. doi: 10.3389/fvets.2025.1509698 (PMC11906662; doi:10.3389/fvets.2025.1509698)
Supplement: Supplementary file 1 [file Data_Sheet_1.zip › Data Sheet 1/Supplementary Figure/Figure S2 Original figures/Figure S2A osa-miR528-5p GOenrichment.pdf]

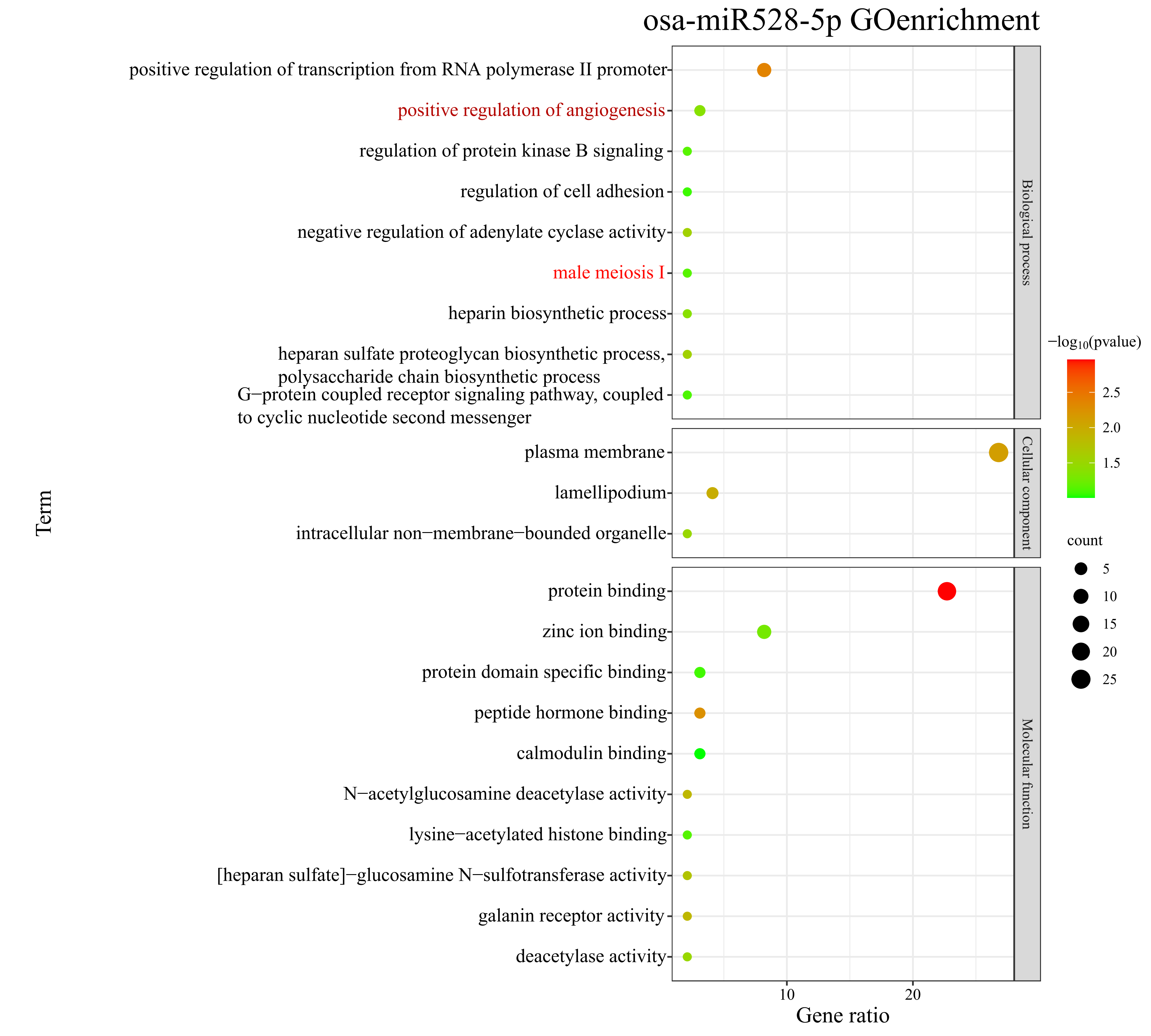
osa-miR528-5p GOenrichment
positive regulation of transcription from RNA polymerase II promoter
positive regulation of angiogenesis
regulation of protein kinase B signaling
regulation of cell adhesion
negative regulation of adenylate cyclase activity
male meiosis I
heparin biosynthetic process
heparan sulfate proteoglycan biosynthetic process, polysaccharide chain biosynthetic process
G−protein coupled receptor signaling pathway, coupled to cyclic nucleotide second messenger
plasma membrane
lamellipodium
intracellular non−membrane−bounded organelle
protein binding
zinc ion binding
protein domain specific binding
peptide hormone binding
calmodulin binding
N−acetylglucosamine deacetylase activity
lysine−acetylated histone binding
[heparan sulfate]−glucosamine N−sulfotransferase activity
galanin receptor activity
deacetylase activity
Biological process
Cellular component
Molecular function
Term
Gene ratio
10
20
$-\log_{10}$(pvalue)
2.5
2.0
1.5
count
5
10
15
20
25
