# Supplementary material for: Cross-kingdom regulation of gene expression in giant pandas via plant-derived miRNA
Source: Front Vet Sci. 2025 Feb 28;12:1509698. doi: 10.3389/fvets.2025.1509698 (PMC11906662; doi:10.3389/fvets.2025.1509698)
Supplement: Supplementary file 1 [file Data_Sheet_1.zip › Data Sheet 1/Supplementary Figure/Figure S3 Original figures/Figure S3B osa-miR528-5pKEGGenrichment.pdf]

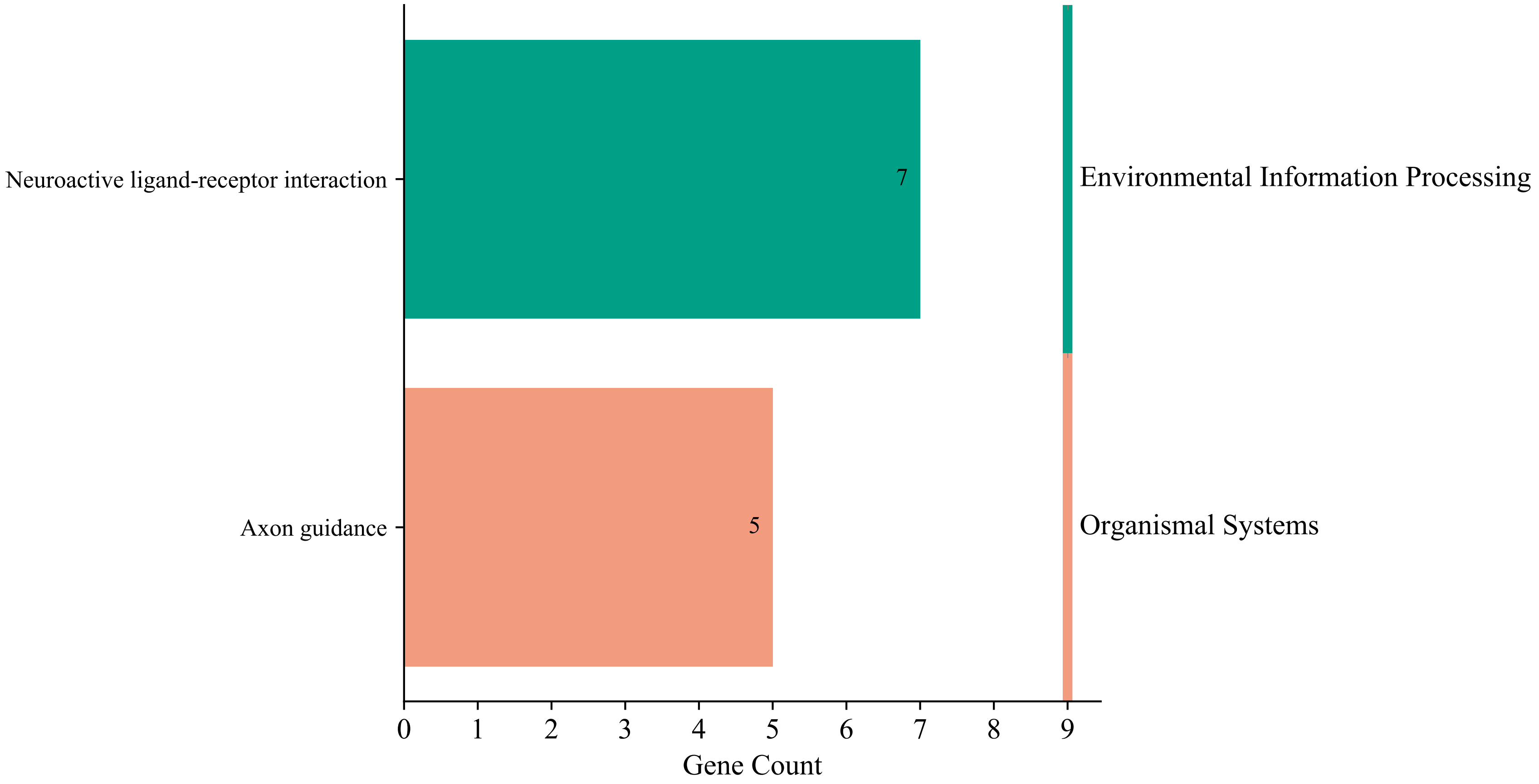
Neuroactive ligand-receptor interaction
Axon guidance
7
5
Environmental Information Processing
Organismal Systems
0
1
2
3
4
5
6
7
8
9
Gene Count
