# Supplementary figures and images for: Cross-kingdom regulation of gene expression in giant pandas via plant-derived miRNA
Source: Front Vet Sci. 2025 Feb 28;12:1509698. doi: 10.3389/fvets.2025.1509698 (PMC11906662; doi:10.3389/fvets.2025.1509698)

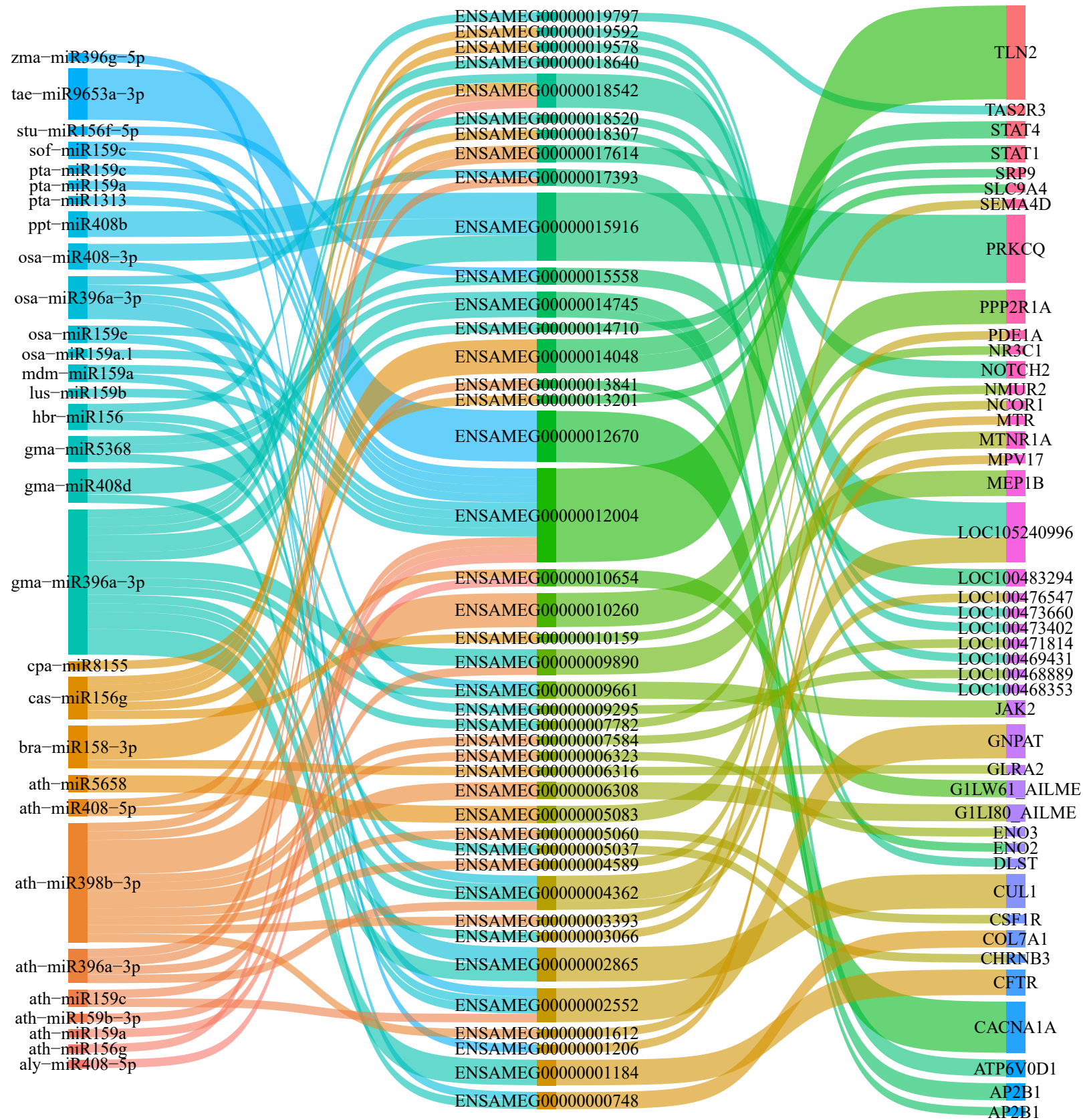

Supplement: Supplementary file 1 [file Data_Sheet_1.zip › Data Sheet 1/Supplementary Figure/Figure S1 Original figures/Figure S1A.pdf]

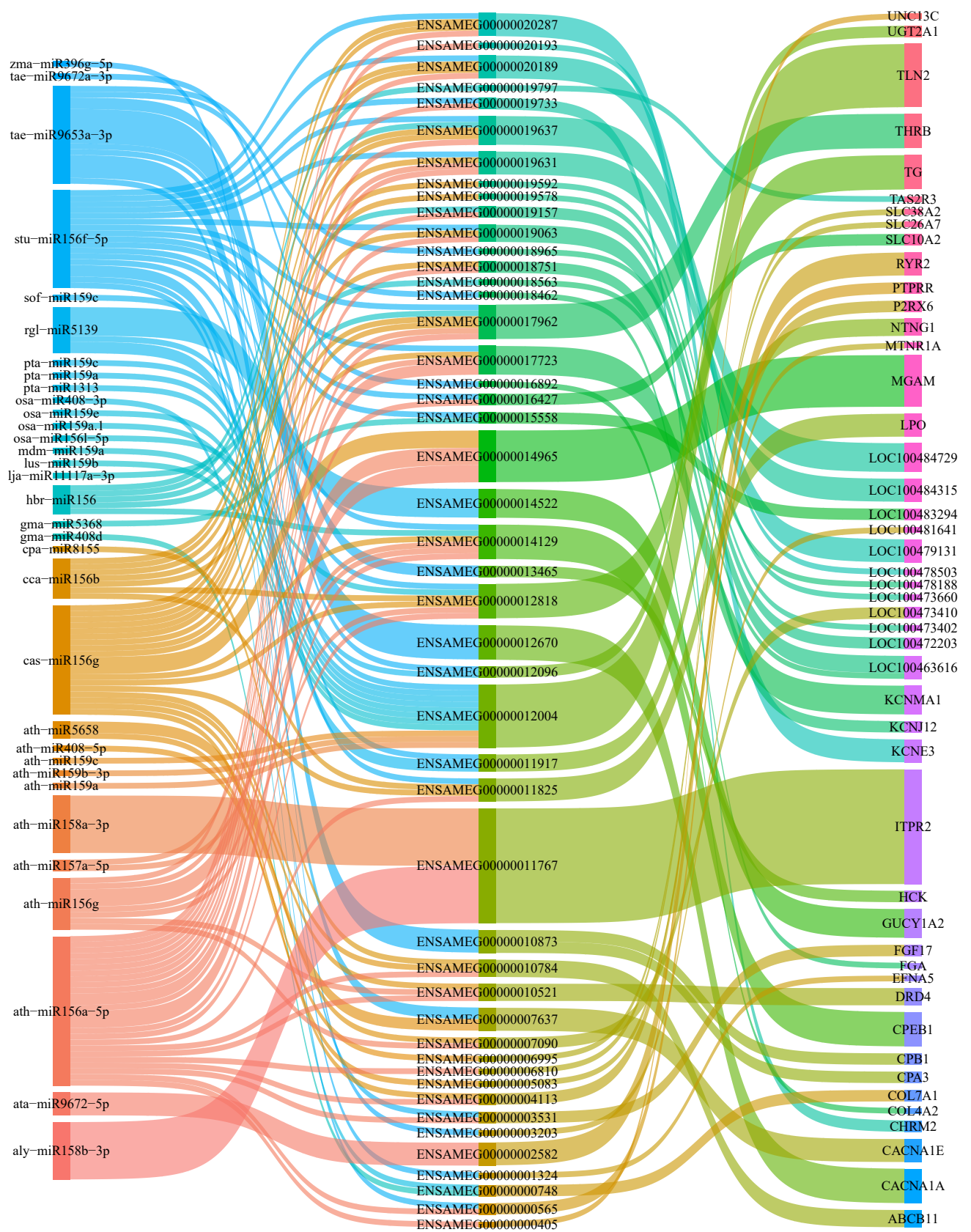

Supplement: Supplementary file 1 [file Data_Sheet_1.zip › Data Sheet 1/Supplementary Figure/Figure S1 Original figures/Figure S1B.pdf]

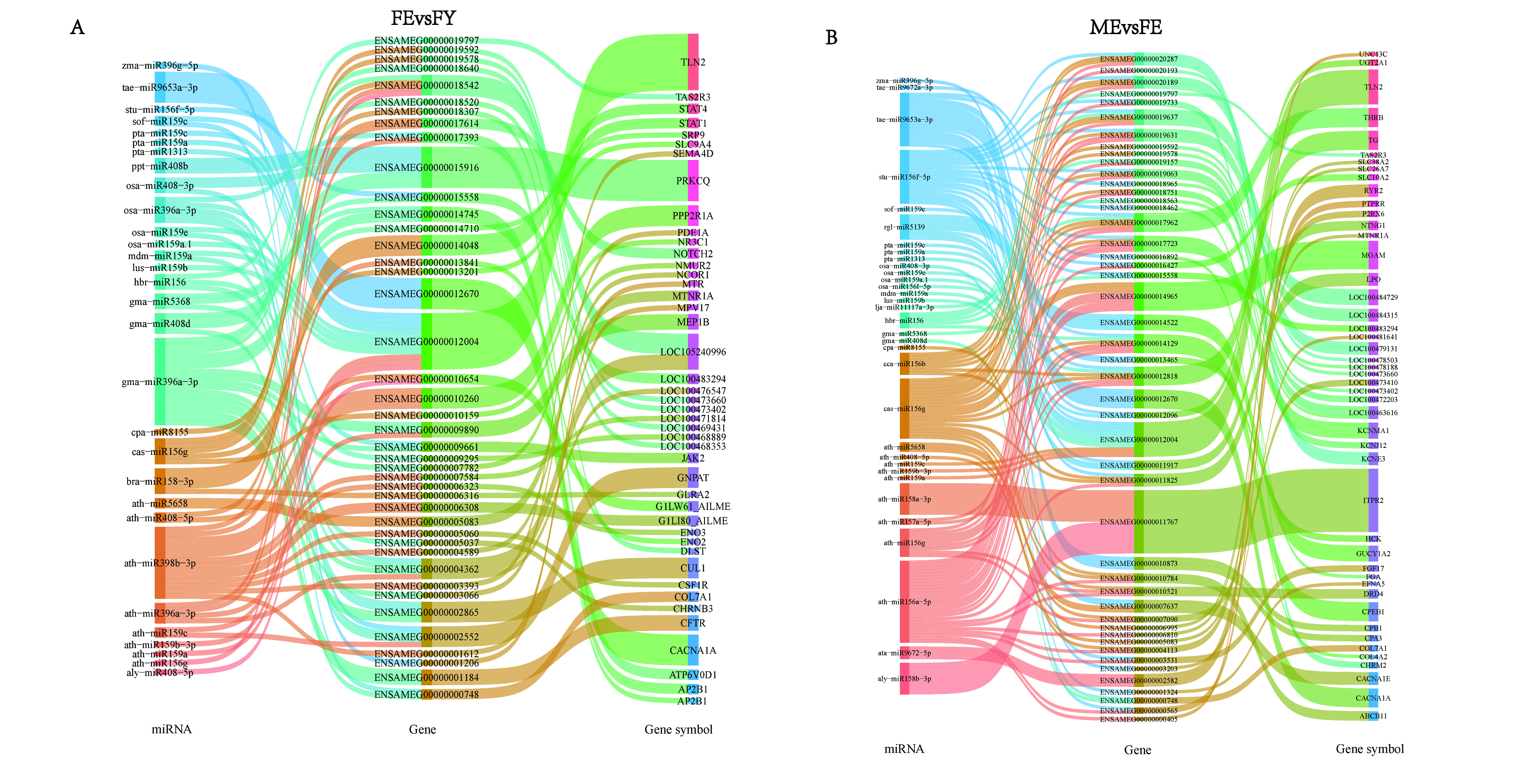

Supplement: Supplementary file 1 [file Data_Sheet_1.zip › Data Sheet 1/Supplementary Figure/Figure S1 Sankey diagram of the relationship between miRNA and corresponding target genes.tif]

# ath-miR159c GOenrichment

Term

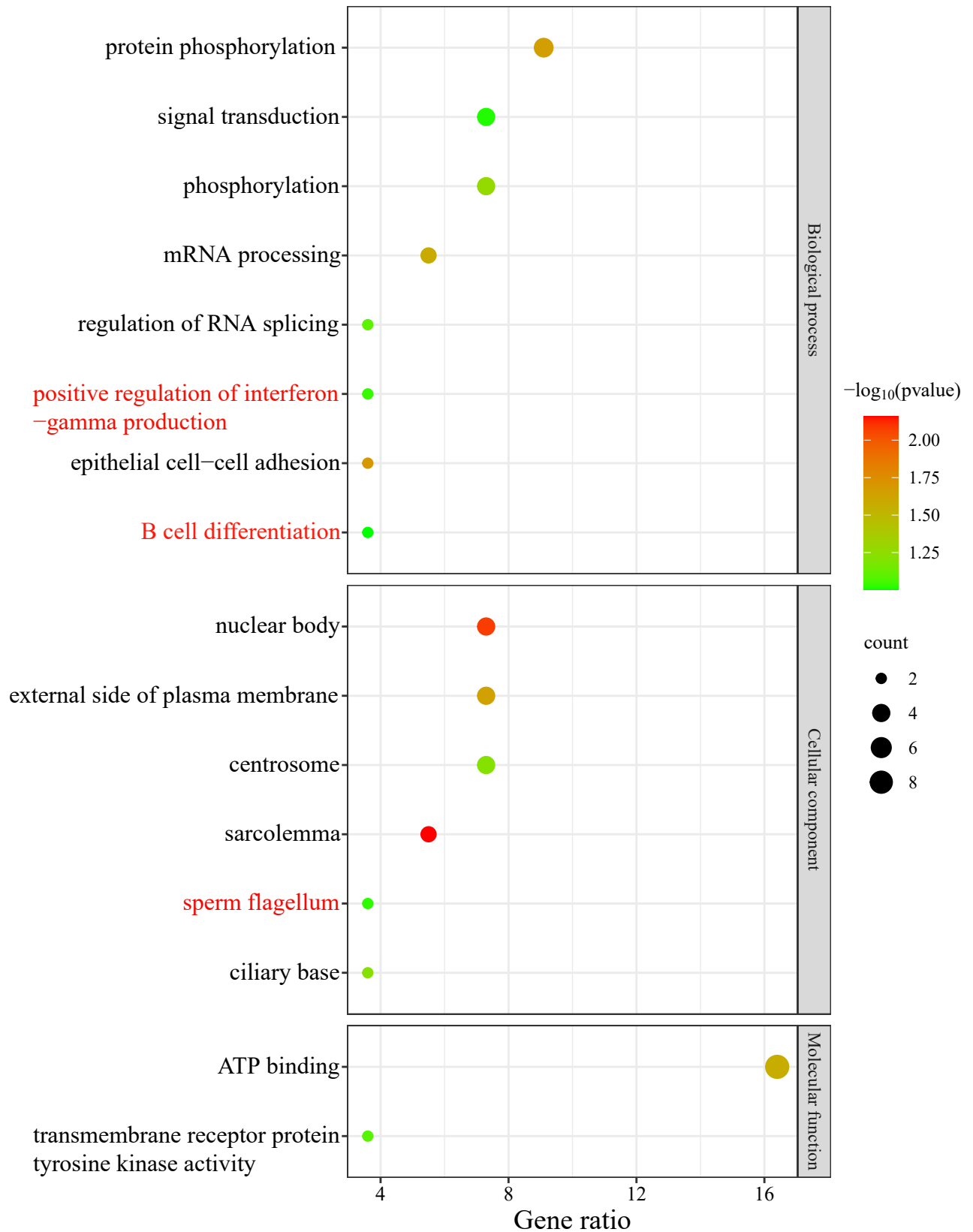

Supplement: Supplementary file 1 [file Data_Sheet_1.zip › Data Sheet 1/Supplementary Figure/Figure S2 Original figures/Figure S2B ath-miR159c GOenrichment.pdf]

# osa-miR166d-5p GOenrichment

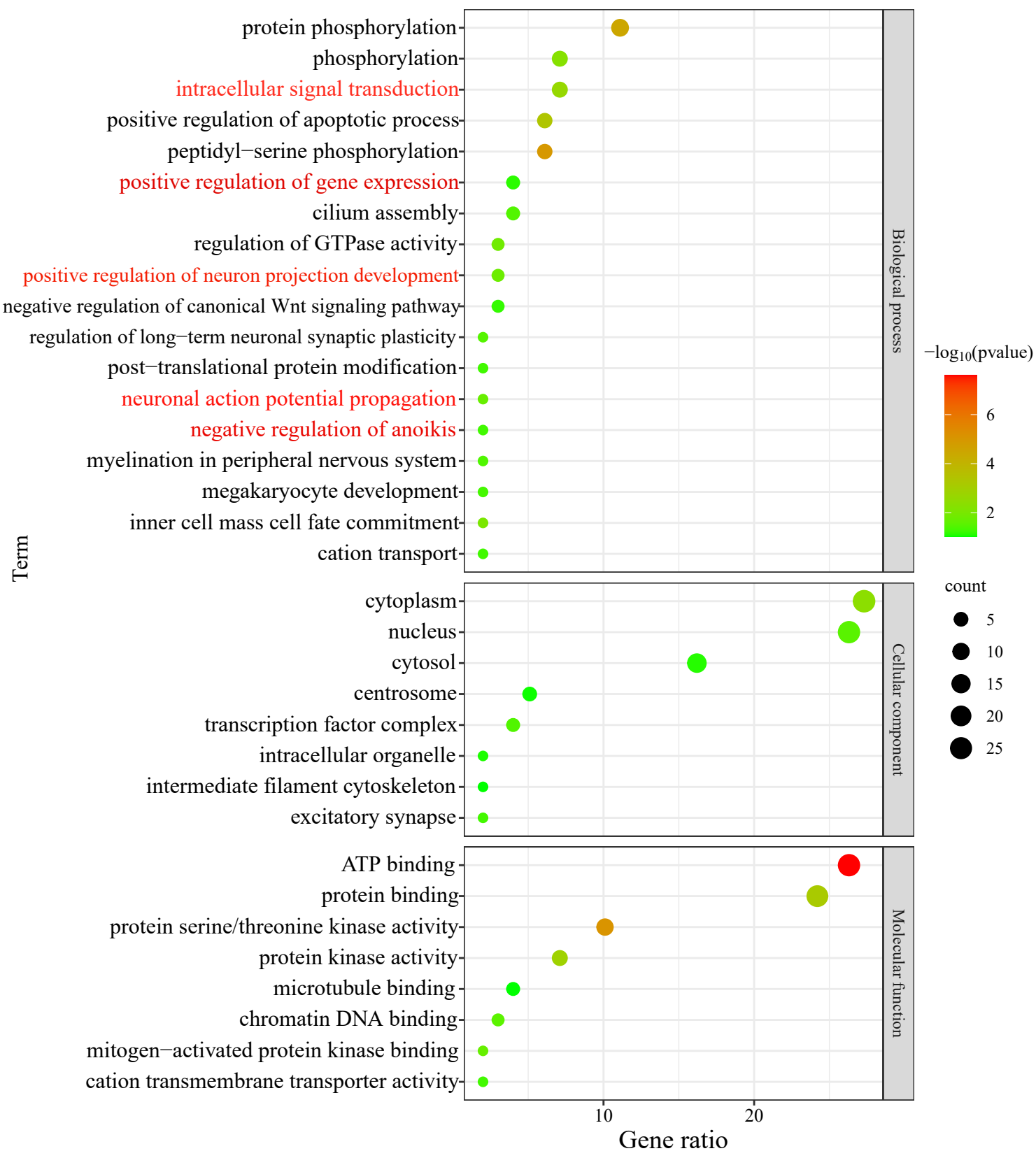

Supplement: Supplementary file 1 [file Data_Sheet_1.zip › Data Sheet 1/Supplementary Figure/Figure S2 Original figures/Figure S2C osa-miR166d-5p GOenrichment.pdf]

# aly-miR158b-3p GOenrichment

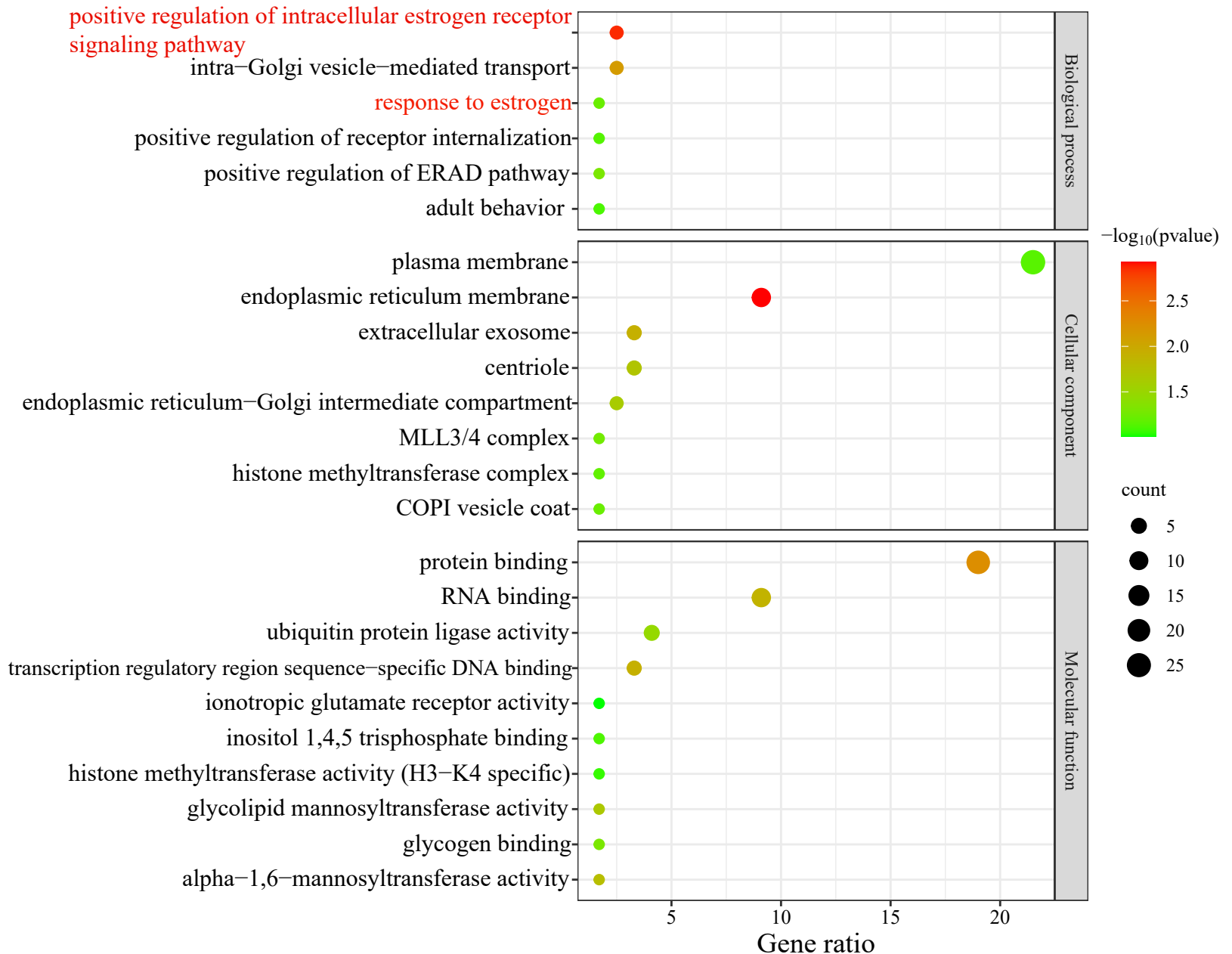

Supplement: Supplementary file 1 [file Data_Sheet_1.zip › Data Sheet 1/Supplementary Figure/Figure S2 Original figures/Figure S2D aly-miR158b-3p GOenrichment.pdf]

# ath-miR159b-3p GOenrichment

Term

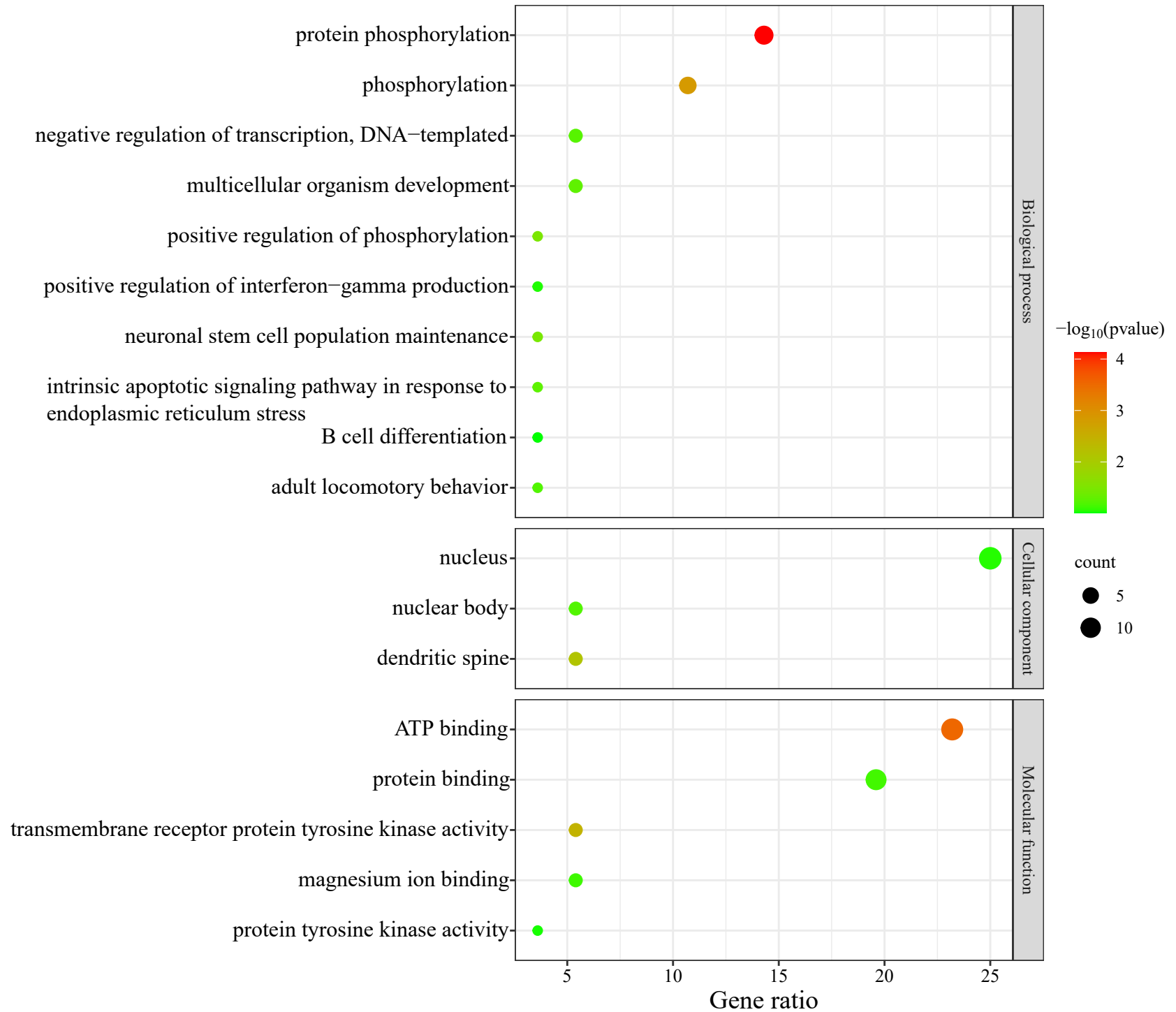

Supplement: Supplementary file 1 [file Data_Sheet_1.zip › Data Sheet 1/Supplementary Figure/Figure S2 Original figures/Figure S2E ath-miR159b-3p GOenrichment.pdf]

# lus-miR159b GOenrichment

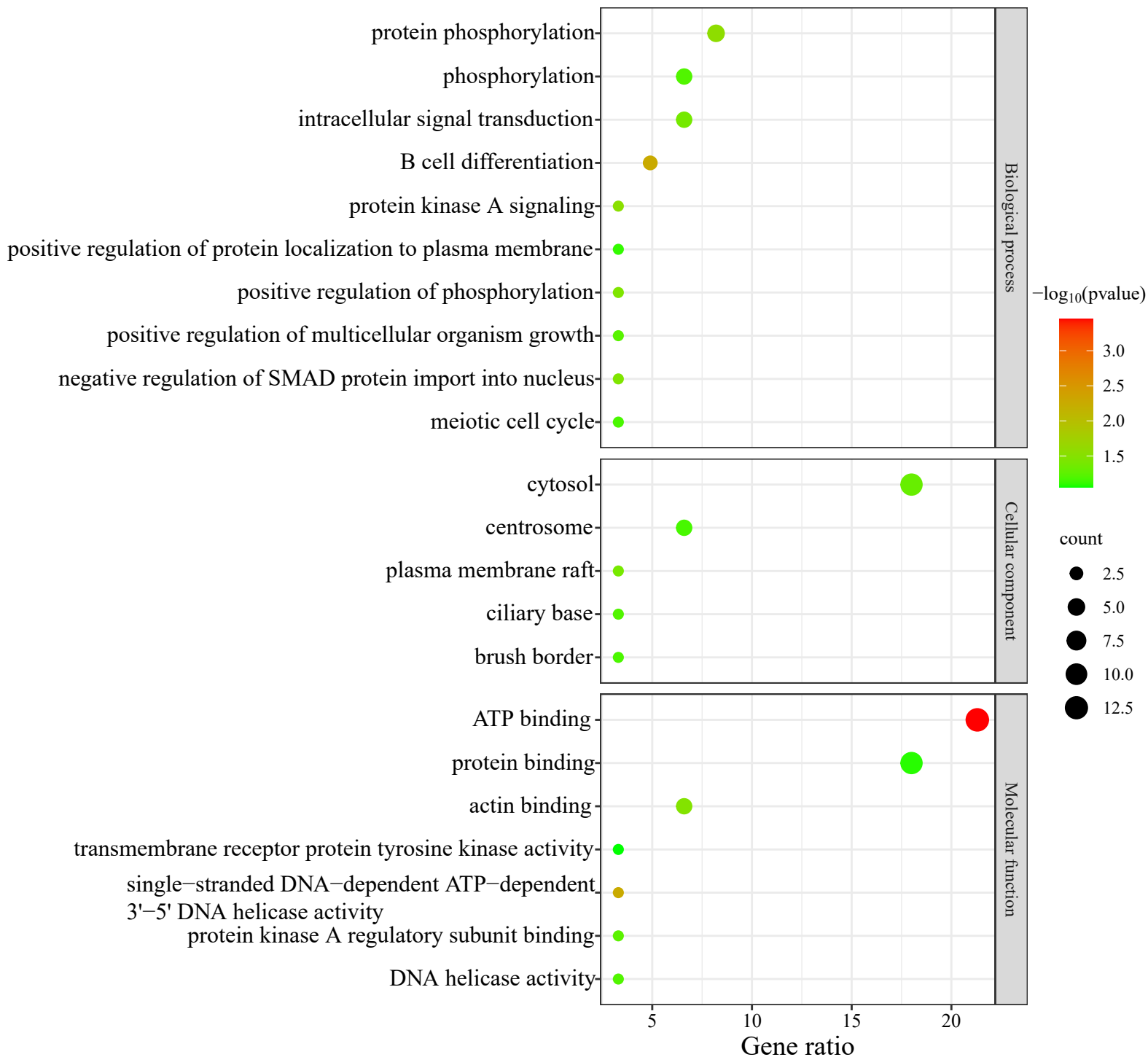

Supplement: Supplementary file 1 [file Data_Sheet_1.zip › Data Sheet 1/Supplementary Figure/Figure S2 Original figures/Figure S2F lus-miR159b GOenrichment.pdf]

# ppt-miR408b GOenrichment

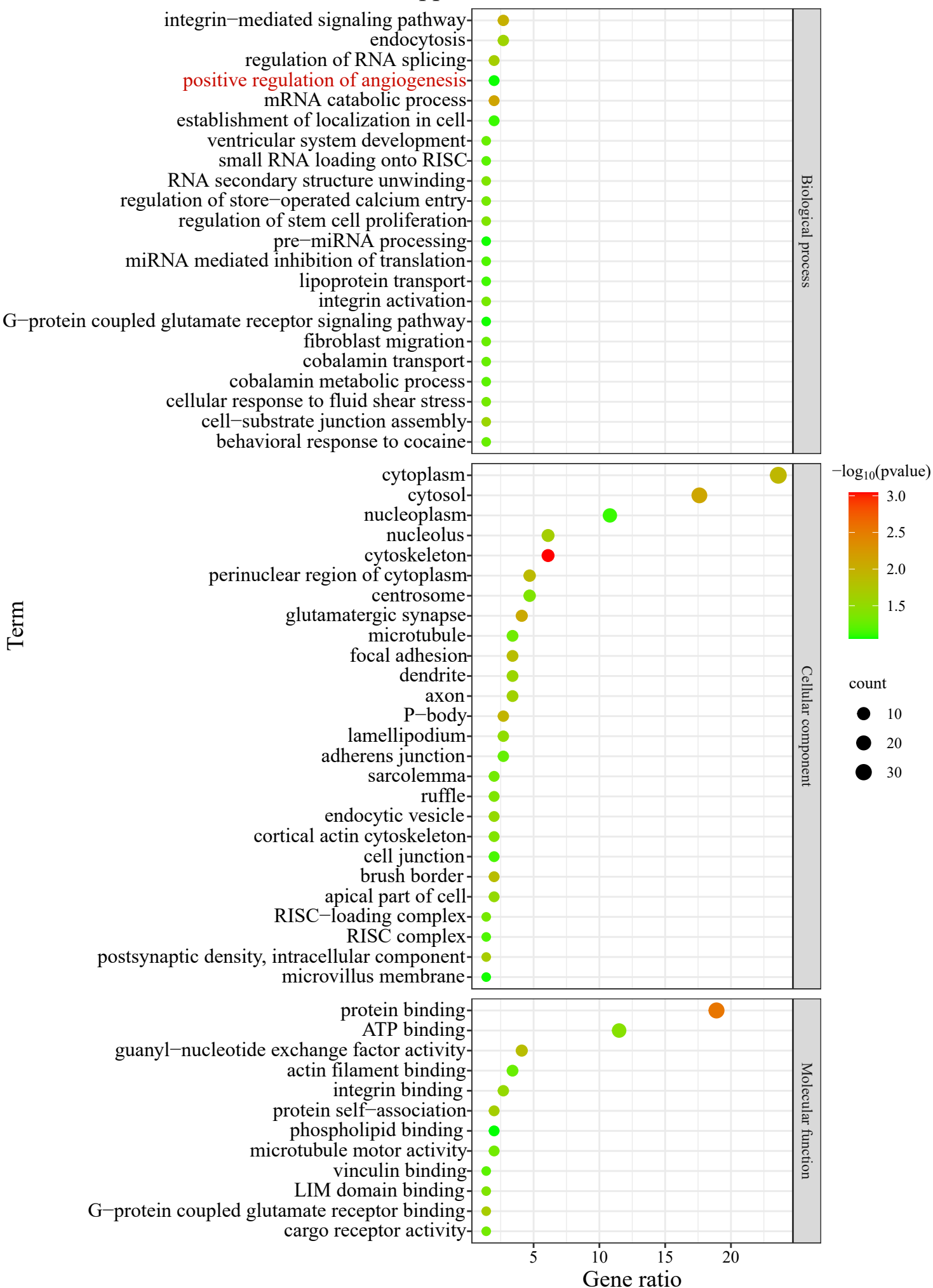

Supplement: Supplementary file 1 [file Data_Sheet_1.zip › Data Sheet 1/Supplementary Figure/Figure S2 Original figures/Figure S2G ppt-miR408b GOenrichment.pdf]

# tae-miR9772 GOenrichment

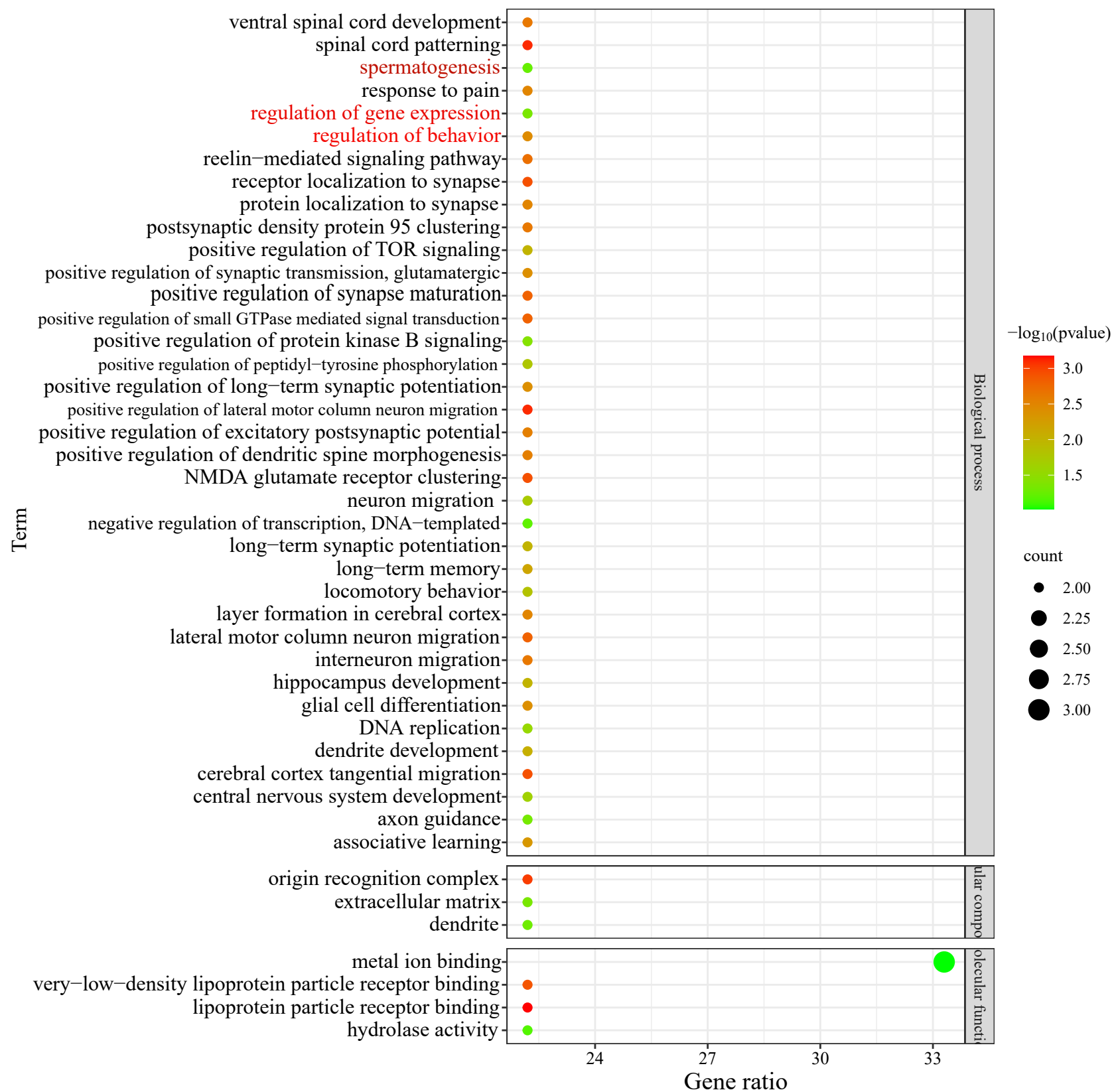

Supplement: Supplementary file 1 [file Data_Sheet_1.zip › Data Sheet 1/Supplementary Figure/Figure S2 Original figures/Figure S2H tae-miR9772 GOenrichment.pdf]

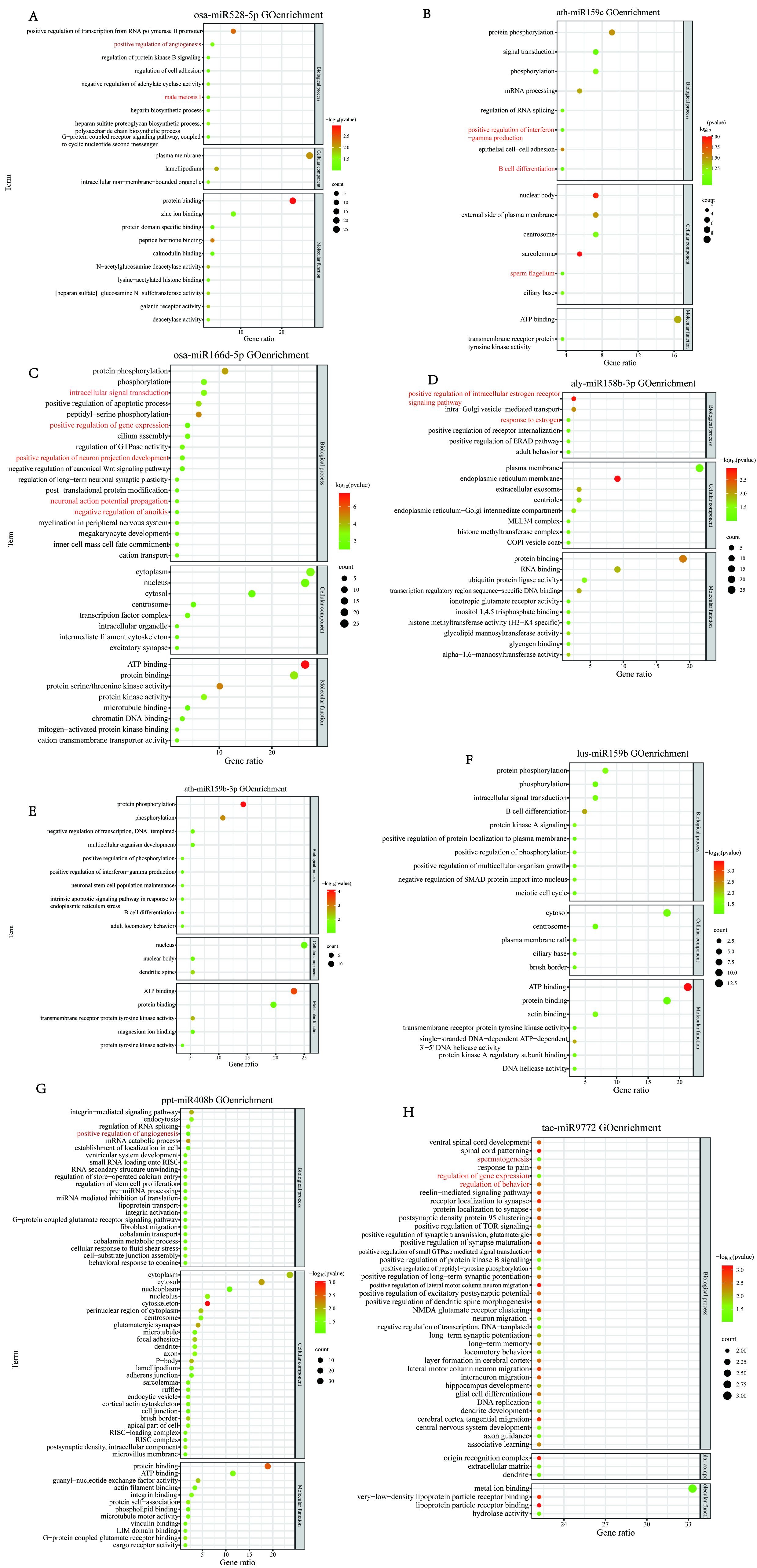

Supplement: Supplementary file 1 [file Data_Sheet_1.zip › Data Sheet 1/Supplementary Figure/Figure S2 Results of GO functional enrichment of miRNAs' target gene..tif]

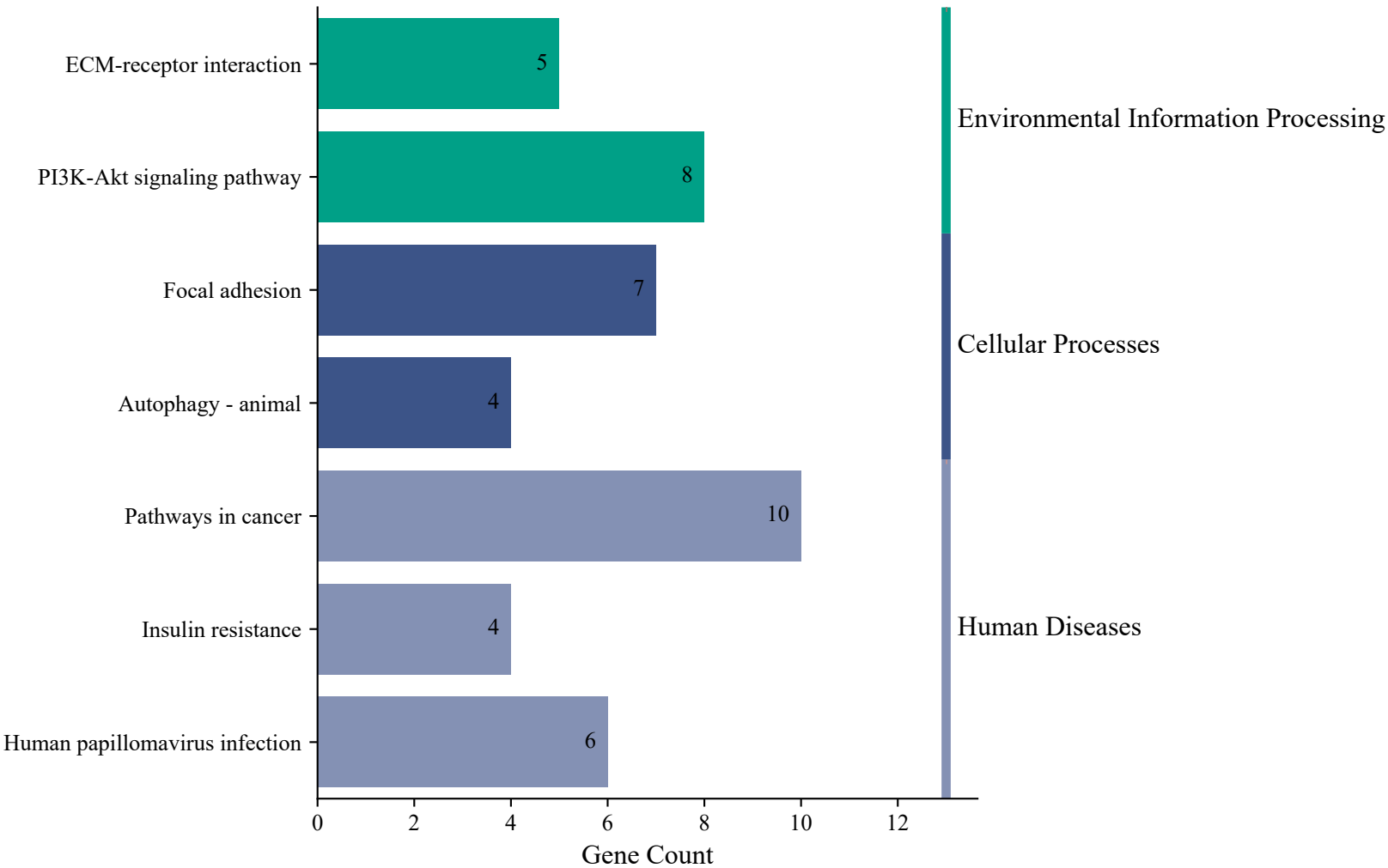

Supplement: Supplementary file 1 [file Data_Sheet_1.zip › Data Sheet 1/Supplementary Figure/Figure S3 Original figures/Figure S3A ppt-miR408bKEGGenrichment.pdf]

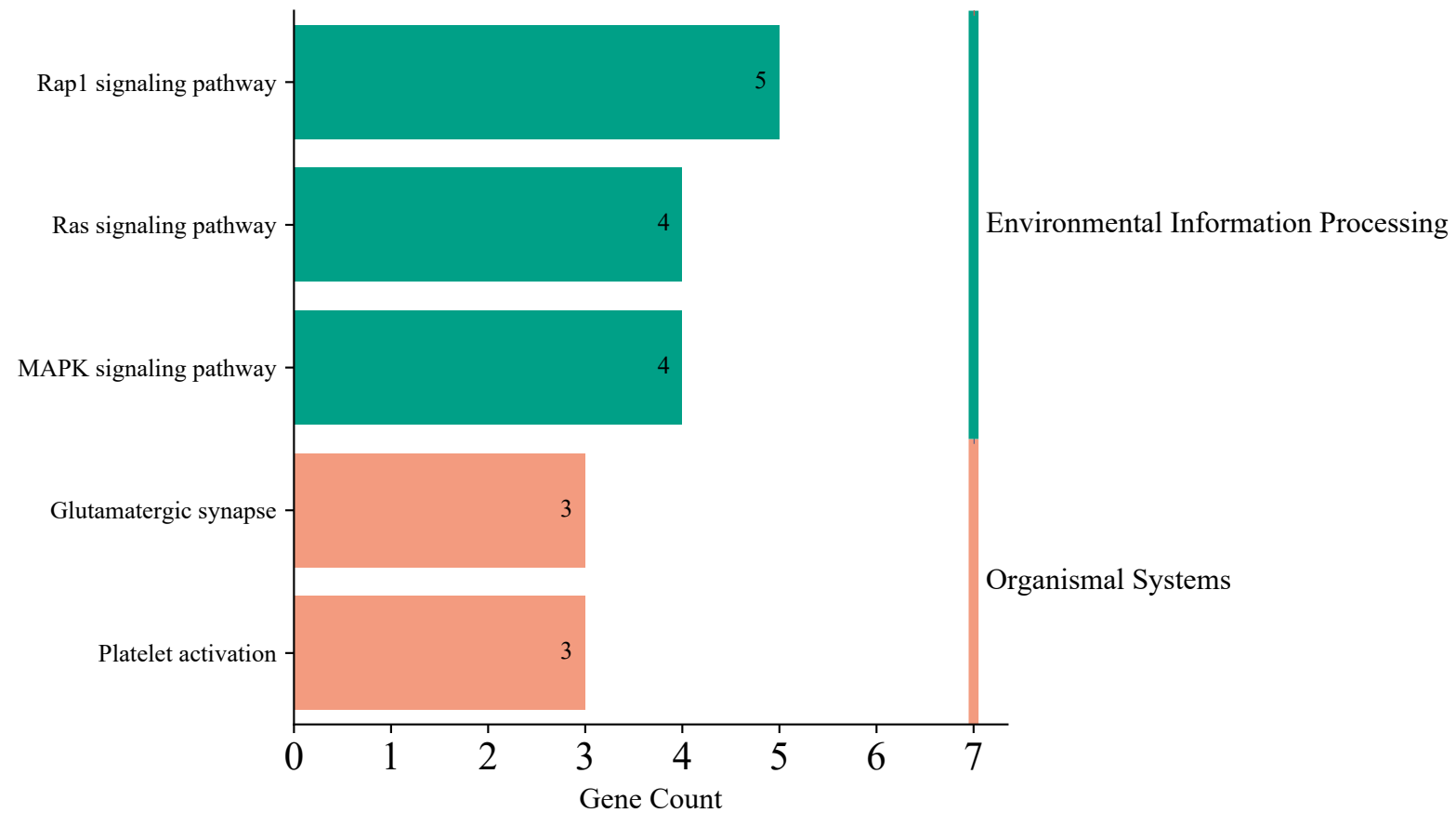

Supplement: Supplementary file 1 [file Data_Sheet_1.zip › Data Sheet 1/Supplementary Figure/Figure S3 Original figures/Figure S3C ath-miR159b-3pKEGGenrichment.pdf]

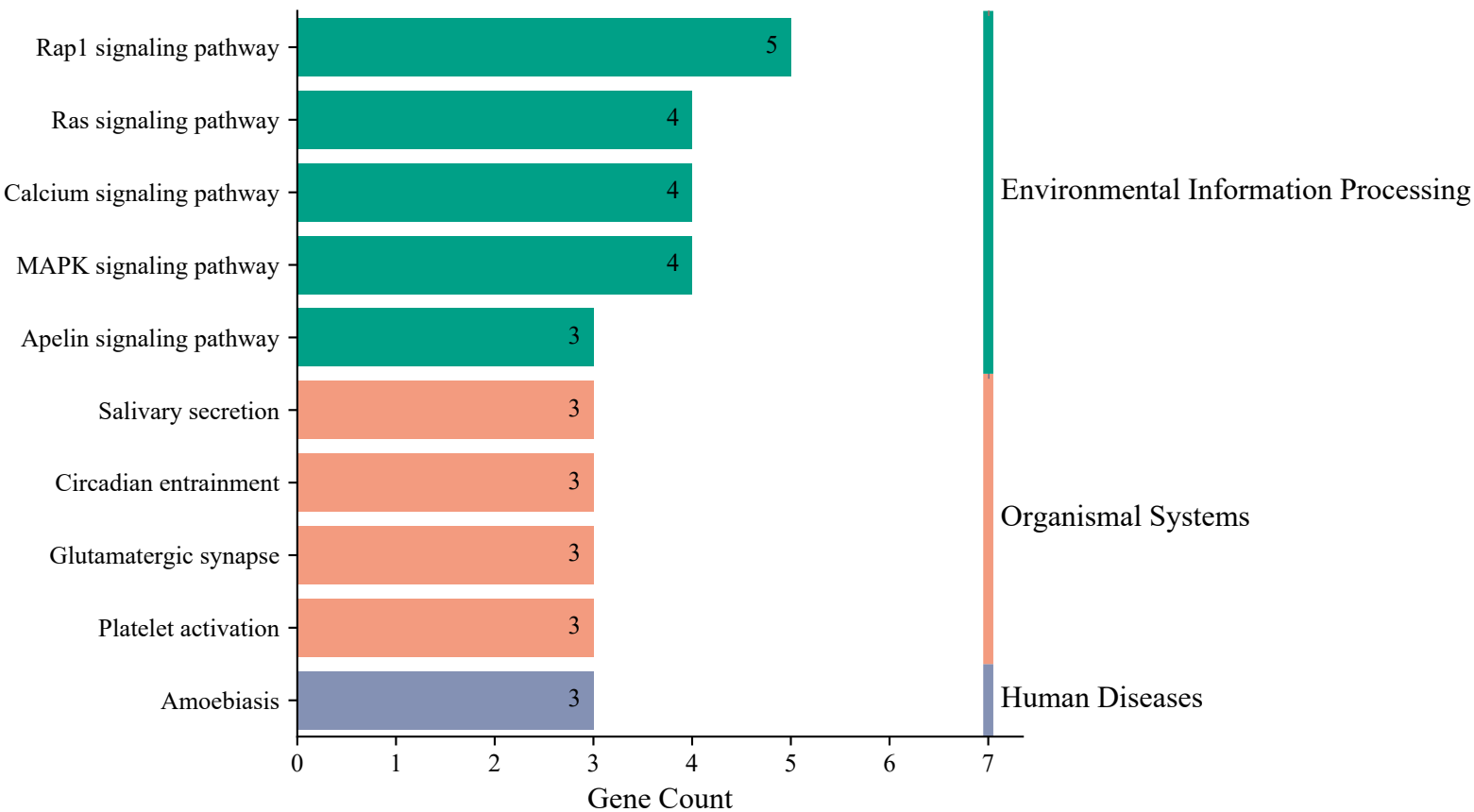

Supplement: Supplementary file 1 [file Data_Sheet_1.zip › Data Sheet 1/Supplementary Figure/Figure S3 Original figures/Figure S3D ath-miR159cKEGGenrichment.pdf]

Fc gamma R-mediated phagocytosis

3

Influenza A

4

Organismal Systems

Human Diseases

0

1

2

3

4

5

6

Gene Count

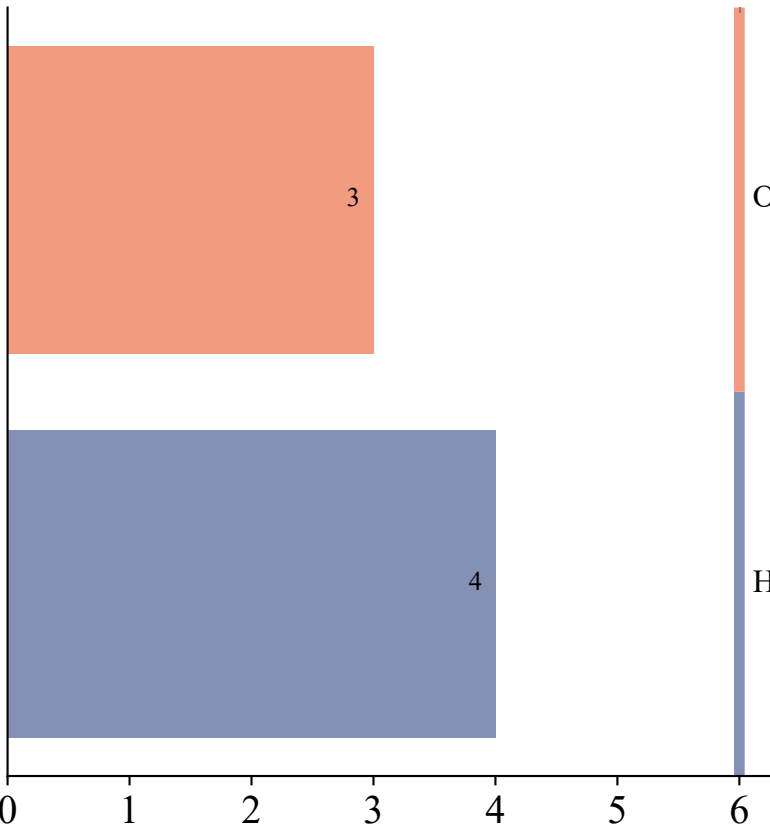

Supplement: Supplementary file 1 [file Data_Sheet_1.zip › Data Sheet 1/Supplementary Figure/Figure S3 Original figures/Figure S3E osa-miR166d-5pKEGGenrichment.pdf]

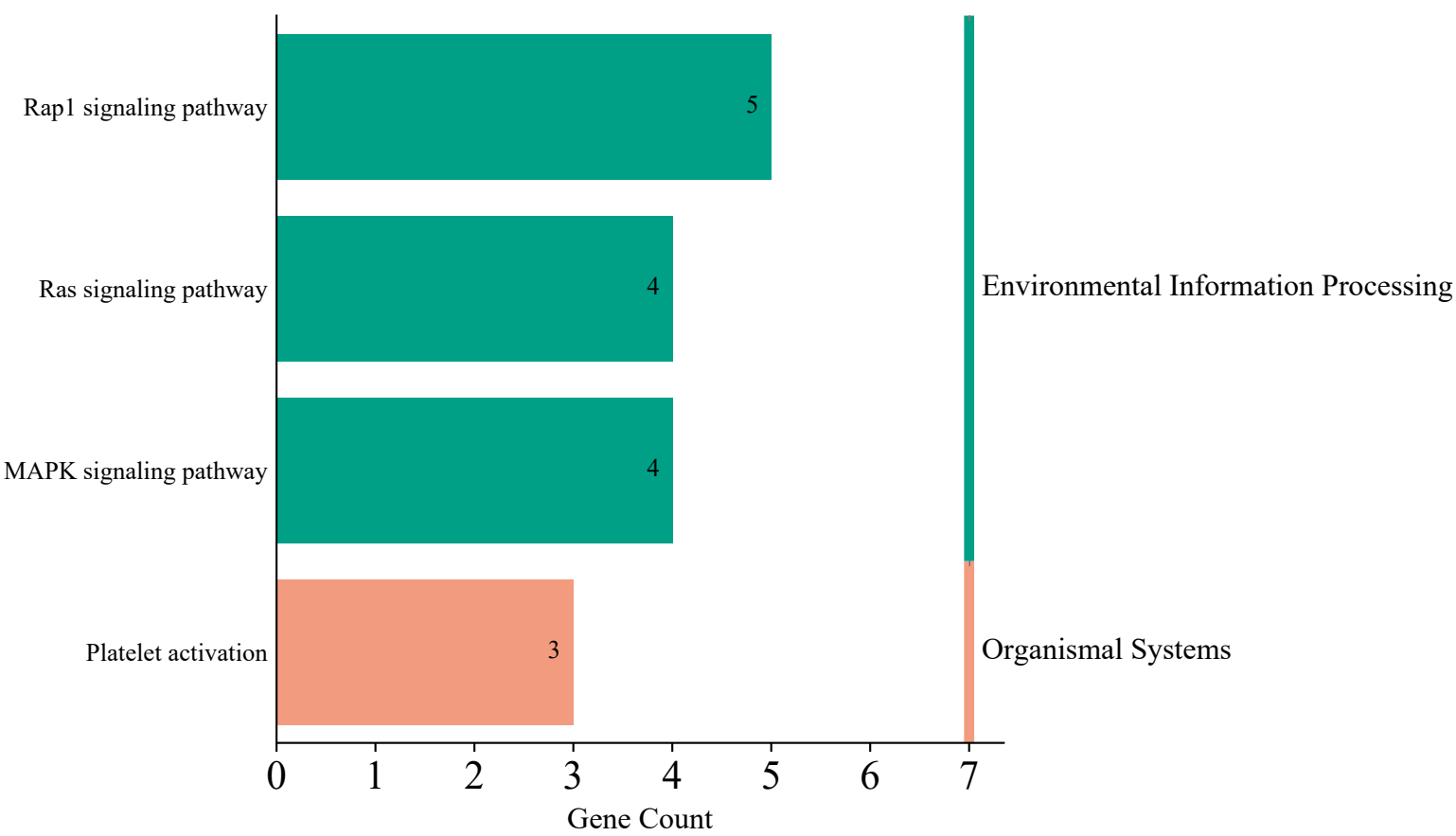

Supplement: Supplementary file 1 [file Data_Sheet_1.zip › Data Sheet 1/Supplementary Figure/Figure S3 Original figures/Figure S3Flus-miR159bKEGGenrichment.pdf]

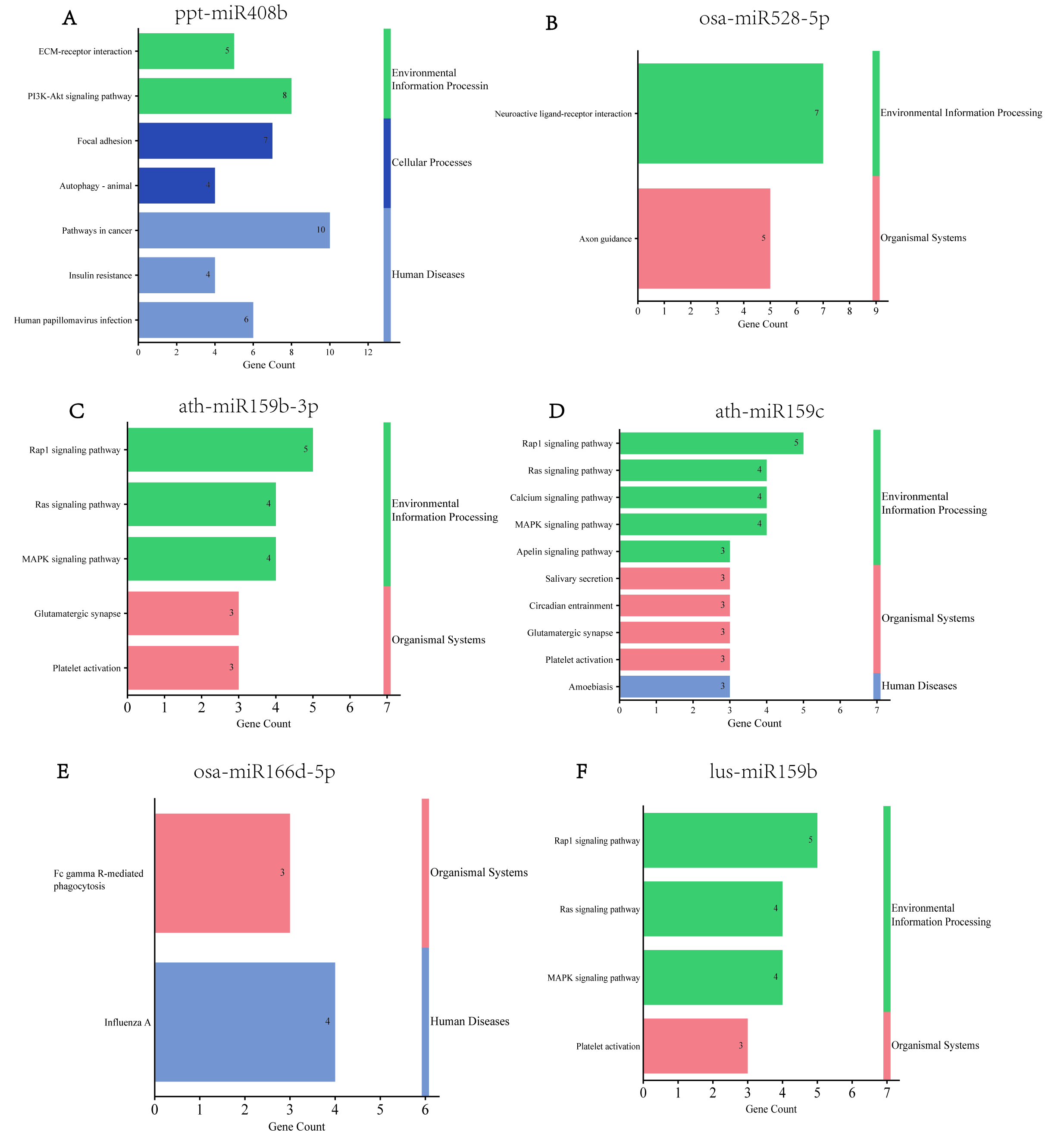

Supplement: Supplementary file 1 [file Data_Sheet_1.zip › Data Sheet 1/Supplementary Figure/Figure S3 Results of KEGG functional enrichment of miRNAs' target gene.tif]
